# Getting a Grip on the Grapevine: Extension and Factor Structure of the Motives to Gossip Questionnaire

**DOI:** 10.3389/fpsyg.2019.01190

**Published:** 2019-05-24

**Authors:** Terence D. Dores Cruz, Daniel Balliet, Ed Sleebos, Bianca Beersma, Gerben A. Van Kleef, Marcello Gallucci

**Affiliations:** ^1^Department of Organization Sciences, Vrije Universiteit Amsterdam, Amsterdam, Netherlands; ^2^Department of Experimental and Applied Psychology, Vrije Universiteit Amsterdam, Amsterdam, Netherlands; ^3^Department of Social Psychology, Universiteit van Amsterdam, Amsterdam, Netherlands; ^4^Department of Psychology, Università Milano-Bicocca, Milan, Italy

**Keywords:** motives to gossip, factorial validity, confirmatory factor analysis, measurement invariance, motives to gossip questionnaire, emotion venting, gossip definition

## Abstract

Gossip is condemned but also ubiquitous and thought to be essential for groups. This triggers the question of which motives explain gossip behavior. Hitherto, negative influence, social enjoyment, group protection, and information gathering and validation are established as motives to gossip. However, venting emotions—discussed as a potentially important motive—has been overlooked empirically. Furthermore, a lack of consensus about a definition of gossip may have affected previous conclusions about gossip motives. This study (*N* = 460) expands the Motives to Gossip Questionnaire (MGQ; Beersma and Van Kleef, 2012) by including a subscale measuring emotion venting, the desire to share emotionally evocative experiences. To validate the five motives to gossip across definitions, we asked participants to report the most recent gossip event they experienced, randomly assigning them to one of three instructions containing different gossip definitions commonly used in the literature: (1) broad instructions (sharing information about third parties who have no knowledge of the exchanged information), (2) narrower instructions (adding that the shared information must be evaluative), and (3) instructions using the word gossip. After participants recalled and described a gossip event, they completed the 25-item measure of five motives to gossip: social enjoyment, information gathering and validation, negative influence, group protection, and emotion venting. Confirmatory factor analysis confirmed the five-factor structure. Multi-group confirmatory factor analysis supported full invariance across the three definition conditions. This indicates the Motives to Gossip Questionnaire successfully measures the five dimensions argued to motivate gossip and can be applied in research conceptualizing gossip both narrowly and broadly.

## Introduction

Gossip is omnipresent across societies, despite being condemned in public opinion (Wilson et al., 2000; Foster, 2004). However, recent literature suggests gossip is essential for social groups because it fosters cooperation and social bonding (e.g., Dunbar, 2004; Nowak and Sigmund, 2005; Wu et al., 2016). The paradoxical nature of gossip as condemned, yet supposedly essential for groups (Dunbar, 2004), raises the question of what motivates people to gossip. The Motives to Gossip questionnaire (MGQ) was developed by Beersma and Van Kleef (2012) to measure four theoretically derived motives: (1) social enjoyment, (2) information gathering and validation, (3) group protection, and (4) social influence (cf. Foster, 2004). The MGQ resolves issues affecting other scales related to gossip; some existing scales assess attitudes toward gossip and gossip use as dispositions, which neglects that gossip motives can differ across situations (Foster, 2004; Litman and Pezzo, 2005). Others solely measure gossip frequency, which does not allow for assessing why people gossip (Nevo et al., 1993; Wittek and Wielers, 1998; Brady et al., 2017).

To our knowledge, the MGQ is the only questionnaire assessing motives to gossip in a specific situation. However, several aspects required improvements. First, despite several authors arguing an important motive to gossip is venting emotions—to share emotionally evocative experiences (e.g., Grosser et al., 2012)—the MGQ lacked a subscale assessing this motive. People have a lay theory of catharsis and this lay theory can regulate their behavior in line with this theory (for example, catharsis beliefs were related to aggressing; Bushman et al., 2001). Whereas the effectiveness of venting emotions via intrapersonal processes is questionable, ample research indicates sharing emotions can elicit responses from others which can contribute to more positive affect (Nils and Rimé, 2012). For instance, people may vent emotions to elicit empathy and support (Pauw et al., 2018b), which are common responses to emotional sharing (Pauw et al., 2018a). Therefore, the desire to vent may motivate gossip about emotional events (Duprez et al., 2015). Indeed, Feinberg et al. (2012) showed reducing negative affect motivated gossip that could successfully alleviate negative affect. Likewise, Brady et al. (2017) found venting emotions was a prominent reason for workplace gossip and Waddington and Fletcher (2005) found nurses gossiped to express emotions. In sum, several studies show venting emotions can be a motive to gossip, suggesting measuring emotion venting is needed.

Second, whereas growing evidence suggests the desire to protect group members against norm violators is a prominent driver of gossip (Beersma and Van Kleef, 2011; Feinberg et al., 2012), this motive was measured with only three items in the MGQ, the minimum number of items to form a scale, making the scale vulnerable to response errors. Therefore, we extended the group protection subscale with two items to increase the robustness of the measure.

Third, for reasons of parsimony, we removed four redundant items from the information gathering and validation scale^1^, such that in the resulting questionnaire, each motive to gossip is measured with five items. Fourth, we carefully checked the wording of existing items to eradicate ambiguities and improve face validity.

Finally, we tested the validity of the MGQ by assessing the invariance of the underlying factor structure of the MGQ against variations in gossip conceptualizations. Gossip has been defined differently across studies. Sometimes it has been broadly defined as information shared about a third party who has no knowledge of the communicated information (cf. Peters and Kashima, 2015). Other studies have used a narrower definition, restricting gossip to information that portrays the target as positive or negative (cf. Foster, 2004), and yet others simply used the word “gossip” (e.g., Jazaieri et al., 2018). Currently, we do not know whether the MGQ enables distinguishing between different motives to gossip across these different conceptualizations. Therefore, we first examined its factorial validity in a representative sample and investigated whether a five-factor structure, distinguishing five motives for gossip as intended, fitted the data best. Second, we examined the measurement invariance of the MGQ across the three gossip definitions.

## Method

### Participants

The total sample comprised a diverse community sample of 493 participants recruited through Dutch panel agency Flycatcher. Thirty-three participants indicated not recalling gossip and were removed. Demographic information was available for 453 participants (50.4% females); age ranged from 18 to 91 (*M* = 46.10, *SD* = 16.86).

### Materials

#### Gossip Definitions

Participants read one of three definitions of gossip in a broad (*N* = 152), narrow (*N* = 155), or simple (*N* = 153) instruction condition. *The broad instructions* instructed participants to report situations in which they communicated information about another person, who was absent or unable to hear the conversation. Participants were informed such information can relate to personal characteristics, attributes, events, behaviors, or needs of this person and could be positive, negative, or neutral. The information could be shared through any medium with any number of people present. Lastly, the information had to concern a specific person and did not have to remain secret to the third person (target of gossip). *The narrow instructions* were identical except for specifying the information must be positive or negative. *The simple instructions* instructed participants to report situations in which they communicated gossip instead of a definition of gossip, which could be shared through any medium and must concern a specific person (Complete instructions reported in Supplementary Materials).

#### Motives to Gossip

We extended the original MGQ by including a five-item subscale measuring emotion venting, adding two items to the group protection subscale, and removing four items from the information gathering and validation subscale^2^, resulting in a 25-item self-report questionnaire measuring five motives to gossip on seven-point Likert scales [1 (*completely disagree*), 7 (*completely agree*)]. Finally, we carefully checked the wording of all items for ambiguities and made minor changes in wording (overview in Supplementary Table 8). Items and descriptive statistics are reported in Supplementary Tables 1–4.

### Procedure

After providing informed consent, participants were randomly assigned to read either the broad, narrow, or simple instructions. Participants described the most recent situation in which they shared gossip (first for the current day, if not, they recalled the next most recent situation). After this, participants completed the MGQ^3^.

### Statistical Analyses

To examine whether the added fifth emotion venting dimension statistically contributes beyond the original four dimensions of the MGQ, we tested the five-factor solution against four-factor solutions using confirmatory factor analysis where emotion venting indicators load on the other dimensions.

We used the R-package Lavaan (Rosseel, 2012; R. Core Team, 2018), applying MLM Robust to correct for substantial multivariate kurtosis (Mardia's coefficients > 159.04, normalized estimates > 21.67) and allowing factors to correlate.

Next, we tested whether factor structures were similar across different gossip definitions by examination of the measurement invariance of the MGQ across the three instructions conditions using the SemTools-package (Hirschfeld and Von Brachel, 2014; Jorgensen et al., 2018). First, we tested for configural invariance (i.e., whether the number of latent variables and the pattern of indicators loading on factors is similar across definitions). Second, we tested for weak (metric) invariance (i.e., whether factor loadings are equal across definitions, indicating indicators reflect the same latent variables with the same intensity across definitions). Third, we tested for strong (scalar) invariance (i.e., whether item intercepts are equal across definitions, indicating the absence of systematic response biases and allowing comparisons of latent variable means across definitions). Fourth, we tested for strict invariance (i.e., equal residual variances across definitions, indicating the indicators show equal explained variance across definitions). Finally, we tested for the equality of factor means across definition conditions.

To assess the five-factor model fit, we used the following criteria for at least a satisfactory fit: CFI > 0.90; TLI > 0.90, RMSEA < 0.08, SRMR < 0.08, χ^2^/*df* < 3.00 (Hu and Bentler, 1999; Hooper et al., 2008). For measurement invariance tests, we used criteria for large samples: *p*-value of < 0.01 for Δχ^2^ (likelihood-ratio test) and ΔCFI> 0.002 indicating lack of measurement invariance (Meade et al., 2008).

## Results

### Factorial Validity

Results showed that, for the complete sample, the five-factor model including a separate emotion venting dimension was satisfactory and significantly better than any four-factor model where the emotion venting items loaded on another latent factor (Table 1).

**Table 1:** Model fit statistics for the five-factor model tested against four-factor models including emotion venting in an existing dimension.

							**Comparison to the five-factor model**			
**Model**	**χ2**	***df***	**Robust CFI**	**Robust TLI**	**Robust RMSEA**	**SRMR**	**Δχ^2^**	**Δ*df***	***p***	**Δ Robust CFI**
Five-Factor	744.40	265	0.926	0.916	0.073	0.079				
Emotion venting in Information gathering and validation	1585.70	269	0.800	0.777	0.119	0.113	928.33	4	< 0.001	−0.126
Emotion venting in Social enjoyment	1842.59	269	0.764	0.737	0.129	0.169	1245.20	4	< 0.001	−0.162
Emotion venting in Negative influence	1652.02	269	0.791	0.767	0.122	0.115	1161.00	4	< 0.001	−0.135
Emotion venting in Group protection	1667.97	269	0.785	0.760	0.124	0.124	1031.50	4	< 0.001	−0.141

*Tested using scaled and shifted test statistics according to Satorra (2000)*.

All standardized factor loadings were acceptable and were statistically significant (*p* < 0.001), ranging from 0.94 to 1.77 (Supplementary Table 1). Correlations were weak to moderately positive and statistically significant (Supplementary Table 5), indicating multiple motives are involved in one gossip situation. This is consistent with a priori assumptions, as there is no reason to expect a specific gossip instance involves a single motive. Furthermore, all subscales showed good internal consistency which did not differ between conditions, indicating the subscales measure their respective dimension across definitions (Supplementary Table 6).

### Measurement Invariance

Indicating configural variance, the model fit was acceptable in all three definition conditions (Supplementary Table 7). All standardized factor loadings were statistically significant (*p* < 0.001), ranging from 0.79 to 1.88 (Supplementary Tables 1–4).

Indicating weak invariance, strong invariance, and strict invariance, imposing the constraints of equal factor loadings, item intercepts, and residual variances did not lead to statistically significant differences in the fit of the five-factor model (Table 2).

**Table 2:** Measurement invariance statistics for the definition conditions.

							**Model difference tests**			
**Type of invariance**	**χ2**	***df***	**Robust CFI**	**Robust TLI**	**Robust RMSEA**	**SRMR**	**Δχ^2^**	**Δ*df***	***p***	**Δ Robust CFI**
Configural	1365.38	795	0.916	0.905	0.079	0.087				
Weak	1401.87	835	0.917	0.911	0.077	0.090	27.12	40	0.940	0.001
Strong	1457.05	875	0.916	0.914	0.075	0.090	47.66	40	0.189	−0.001
Strict	1492.79	925	0.915	0.917	0.074	0.090	55.49	50	0.278	−0.001
Factor means	1513.38	935	0.914	0.917	0.074	0.092	24.71	10	0.006	−0.001

*Tested using scaled and shifted test statistics according to Satorra (2000)*.

Imposing the constraint of equal factor means led to a significantly worse model fit (Table 2), indicating at least two of the conditions differed on at least one factor. Comparing all factor means across conditions showed the participants in the simple condition scored higher on negative influence (Δ*M*_*broad*_ = 0.30, *p* = 0.033) compared to the broad condition and higher than broad and narrow conditions on information gathering and validation (Δ*M*_*broad*_ = 0.32, *p* = 0.039; Δ*M*_*narrow*_ = 0.34, *p* = 0.029). It is possible that the gossip condition fits better with lay views of gossip as negative and informative about others. However, ΔCFI did not exceed the 0.002 criterion. Given the ΔCFI and the large sample, this difference among means does not seem important or very reliable (supported by a MANOVA testing mean differences for all motives per condition [Pillai's Trace = 0.35, *F*_(10, 908)_ = 1.61, *p* = 0.099].

In summary, across definitions, the MGQ reliably measures gossip motives that can be clearly distinguished, despite positive inter-correlations.

## Discussion

The five-factor Motives to Gossip model showed satisfactory fit and good reliability across subscales, indicating the revised MGQ successfully distinguishes social enjoyment, information gathering and validation, negative influence, group protection, and emotion venting motives to gossip. Results support Beersma and Van Kleef's (2012) theoretical model of gossip motives and extend it by including emotion venting. Enabling the measurement of this motive enhances the MGQ's ability to assess why people gossip.

Furthermore, the MGQ factor structure was completely invariant across broad, narrow, and simple definitions of gossip, indicating the MGQ is a stable instrument unaffected by different gossip definitions, which is corroborated by the dimensions' equal internal consistency across definitions. This implicates the MGQ can be used regardless of how gossip is defined. Furthermore, this enables comparing results across studies such as in meta-analyses.

### Limitations and Strengths

Our study also had limitations. Firstly, we do not consider criterion variables such as gossip frequency or intensity, therefore it remains unclear whether emotion venting improves the predictive validity of the MGQ and whether the MGQ motives can predict criteria similarly across gossip definitions. Therefore, future research should investigate the MGQ's predictive validity for relevant criterion variables. Secondly, we did not consider contextual variables (e.g., group characteristics, Grosser et al., 2010, or personality, Lyons and Hughes, 2015) which could influence the MGQ's functioning.

Despite these limitations, our study is unique in its focus on measurement invariance across gossip definitions. We contribute to both understanding gossip motives and integrating gossip literature. Moreover, we used a large, diverse sample. This affords adequate power and allows generalizability beyond student or employee samples commonly used in gossip research. Lastly, asking respondents to report on real gossip events shows the motives captured in the MGQ are important in the natural setting where gossip occurs.

## Conclusion

We demonstrated the MGQ is a stable and reliable instrument capturing several motives underlying gossip and aids our understanding of why people engage in the sometimes-puzzling behavior of gossip.

## Ethics Statement

This study was carried out in accordance with the recommendations of Code of Ethics for Research in the Social and Behavioral Sciences, Scientific and Ethical Review Board (VCWE) of the Faculty of Behavioral and Movement Sciences at the VU University Amsterdam with written informed consent from all subjects. All subjects gave written informed consent in accordance with the Declaration of Helsinki. The protocol was approved by the Scientific and Ethical Review Board (VCWE).

## Author Contributions

BB and GvK developed the MGQ. TD and DB collected the data. TD and ES analyzed the data. TD wrote the first draft of the manuscript. DB, ES, BB, GvK, MG, and TD commented and worked on sections resulting in the current manuscript.

### Conflict of Interest Statement

The authors declare that the research was conducted in the absence of any commercial or financial relationships that could be construed as a potential conflict of interest.

## Footnotes

^1^Results did not differ using all items.

^2^An earlier adaptation of the MGQ (Dores Cruz et al., under review) measured emotion venting with seven items. For parsimony and because factor analysis demonstrated 2 items were redundant, we excluded two items from this earlier adaptation to create the current five-item subscale. Results did not differ using all items.

^3^Included in larger survey, see Supplementary Materials.
