# Supplementary material for: Getting a Grip on the Grapevine: Extension and Factor Structure of the Motives to Gossip Questionnaire
Source: Front Psychol. 2019 May 24;10:1190. doi: 10.3389/fpsyg.2019.01190 (PMC6543765; doi:10.3389/fpsyg.2019.01190)
Supplement: Supplementary file 1 [file Table_1.docx]

Supplementary Material

# Supplementary Tables

## Supplementary Table 1

Factor loadings and descriptive statistics for the MGQ across definition conditions.

| MGQ Factor | Standardized Loading (*SE*) | *M* | *SD* |
| --- | --- | --- | --- |
| Emotion venting |  | 3.78 | 1.63 |
| 1. I wanted to get something off my chest. | 1.56 (0.07) | 4.30 | 1.93 |
| 1. I wanted to vent. | 1.64 (0.06) | 3.52 | 2.02 |
| 1. I wanted to resolve my feelings by talking. | 1.66 (0.06) | 4.10 | 1.95 |
| 1. I wanted to I wanted to let off steam. | 1.64 (0.06) | 3.84 | 1.87 |
| 1. I hoped it would make me feel better. | 1.13 (0.07) | 3.13 | 1.84 |
| Information gathering and validation |  | 3.95 | 1.68 |
| 1. I wanted to find out if the recipient felt the same about the third person as I did. | 1.31 (0.07) | 4.30 | 1.79 |
| 1. I wanted to find out whether my impression of the third person was accurate. | 1.60 (0.07) | 3.90 | 1.98 |
| 1. I wanted to find out whether the recipient had the same ideas about the third person as I did. | 1.59 (0.06) | 4.18 | 1.85 |
| 1. I wanted to find out if I thought the same way about the third person as the receiver. | 1.62 (0.06) | 4.00 | 1.92 |
| 1. I wanted to find out whether my ideas about the third person were accurate. | 1.61 (0.06) | 3.80 | 1.94 |
| Social enjoyment |  | 3.48 | 1.46 |
| 1. It was a pleasant pastime. | 0.94 (0.08) | 3.62 | 1.64 |
| 1. I wanted to have a good time with the recipient. | 1.42 (0.06) | 3.46 | 1.75 |
| 1. Talking like this was a pleasant activity. | 1.40 (0.07) | 3.74 | 1.75 |
| 1. I wanted to have fun spending time with the recipient. | 1.59 (0.05) | 3.32 | 1.74 |
| 1. I wanted to have a pleasant time with the recipient. | 1.61 (0.05) | 3.26 | 1.80 |
| Negative influence |  | 2.37 | 1.32 |
| 1. I wanted to damage the third person’s reputation. | 1.20 (0.06) | 1.99 | 1.45 |
| 1. I wanted to speak ill of the third person. | 1.23 (0.06) | 2.09 | 1.47 |
| 1. I wanted to negatively influence the recipient’s impression of the third person. | 1.31 (0.06) | 2.22 | 1.54 |
| 1. I wanted to discredit the third person. | 1.38 (0.05) | 2.13 | 1.50 |
| 1. I wanted to discuss the third person’s negative characteristics. | 1.15 (0.07) | 3.43 | 1.98 |
| Group protection |  | 2.98 | 1.63 |
| 1. I wanted to protect the recipient from the third person’s behavior. | 1.55 (0.07) | 2.74 | 1.88 |
| 1. I wanted to ensure that the recipient would know what kind of person the third person is. | 1.06 (0.08) | 3.54 | 1.90 |
| 1. I wanted to warn the receiver for the third person’ behavior. | 1.68 (0.06) | 2.91 | 1.92 |
| 1. I wanted to make sure that the recipient was not victimized by the third person’s behavior. | 1.74 (0.05) | 2.84 | 1.92 |
| 1. I wanted to prevent the recipient from becoming a victim of the third person’s behavior. | 1.77 (0.05) | 2.89 | 1.92 |

*Note.* Each item starts with the stem “For me, a reason to initiate this communication was …”. The latent factors were standardized, allowing free estimation of all factor loadings. All items were statistically significant at *p* < .001. Items were back-translated from Dutch and any discrepancies were solved through discussion between the first and second author. Dutch items are available through contacting the first author.

## Supplementary Table 2

Factor loadings and descriptive statistics for the MGQ in the broad definition condition.

| MGQ Factor | Standardized Loading (*SE*) | *M* | *SD* |
| --- | --- | --- | --- |
| Emotion venting |  | 3.64 | 1.62 |
| 1. I wanted to get something off my chest. | 1.47 (0.12) | 4.14 | 1.97 |
| 1. I wanted to vent. | 1.67 (0.10) | 3.44 | 2.04 |
| 1. I wanted to resolve my feelings by talking. | 1.67 (0.11) | 3.97 | 2.02 |
| 1. I wanted to share my feelings. | 1.67 (0.10) | 3.72 | 1.90 |
| 1. I hoped it would make me feel better. | 1.21 (0.12) | 2.93 | 1.73 |
| Information gathering and validation |  | 3.83 | 1.74 |
| 1. I wanted to compare my ideas about the third person with the recipient. | 1.31 (0.12) | 4.26 | 1.77 |
| 1. I wanted to find out whether my impression of the third person was accurate. | 1.57 (0.12) | 3.86 | 1.99 |
| 1. I wanted to find out whether the recipient had the same ideas about the third person as I did. | 1.66 (0.11) | 4.04 | 1.93 |
| 1. I wanted to find out if I thought the same way about the third person as the receiver. | 1.63 (0.12) | 3.89 | 1.99 |
| 1. I wanted to find out whether my ideas about the third person were accurate. | 1.66 (0.10) | 3.65 | 1.97 |
| Social enjoyment |  | 3.59 | 1.54 |
| 1. It was a pleasant pastime. | 0.96 (0.13) | 3.65 | 1.63 |
| 1. I wanted to have a good time with the recipient. | 1.48 (0.11) | 3.57 | 1.80 |
| 1. Talking like this was a pleasant activity. | 1.52 (0.11) | 3.90 | 1.87 |
| 1. I wanted to have fun spending time with the recipient. | 1.69 (0.08) | 3.38 | 1.80 |
| 1. I wanted to have a pleasant time with the recipient. | 1.70 (0.09) | 3.43 | 1.87 |
| Negative influence |  | 2.19 | 1.31 |
| 1. I wanted to damage the third person’s reputation. | 1.24 (0.12) | 1.86 | 1.44 |
| 1. I wanted to speak ill of the third person. | 1.22 (0.12) | 1.91 | 1.39 |
| 1. I wanted to negatively influence the recipient’s impression of the third person. | 1.28 (0.12) | 2.09 | 1.52 |
| 1. I wanted to discredit the third person. | 1.31 (0.11) | 1.91 | 1.40 |
| 1. I wanted to discuss the third person’s negative characteristics. | 1.18 (0.12) | 3.18 | 2.00 |
| Group protection |  | 2.82 | 1.55 |
| 1. I wanted to protect the recipient from the third person’s behavior. | 1.45 (0.12) | 2.64 | 1.82 |
| 1. I wanted to ensure that the recipient would know what kind of person the third person is. | 1.08 (0.14) | 3.24 | 1.90 |
| 1. I wanted to warn the receiver for the third person’ behavior. | 1.55 (0.11) | 2.70 | 1.82 |
| 1. I wanted to make sure that the recipient was not victimized by the third person’s behavior. | 1.62 (0.11) | 2.72 | 1.92 |
| 1. I wanted to prevent the recipient from becoming a victim of the third person’s behavior. | 1.63 (0.08) | 2.79 | 1.89 |

*Note.* Each item starts with the stem “For me, a reason to initiate this communication was …”. The latent factors were standardized, allowing free estimation of all factor loadings. All items were statistically significant at *p* < .001.

## Supplementary Table 3

Factor loadings and descriptive statistics for the MGQ in the narrow definition condition.

| MGQ Factor | Standardized Loading (*SE*) | *M* | *SD* |
| --- | --- | --- | --- |
| Emotion venting |  | 3.72 | 1.65 |
| 1. I wanted to get something off my chest. | 1.76 (0.10) | 4.18 | 1.99 |
| 1. I wanted to vent. | 1.61 (0.11) | 3.42 | 2.03 |
| 1. I wanted to resolve my feelings by talking. | 1.68 (0.10) | 4.02 | 1.93 |
| 1. I wanted to share my feelings. | 1.60 (0.11) | 3.80 | 1.86 |
| 1. I hoped it would make me feel better. | 1.18 (0.13) | 3.17 | 1.80 |
| Information gathering and validation |  | 3.78 | 1.64 |
| 1. I wanted to find out if the recipient felt the same about the third person as I did. | 1.33 (0.12) | 4.27 | 1.72 |
| 1. I wanted to find out whether my impression of the third person was accurate. | 1.65 (0.10) | 3.70 | 1.93 |
| 1. I wanted to find out whether the recipient had the same ideas about the third person as I did. | 1.54 (0.11) | 4.08 | 1.79 |
| 1. I wanted to find out if I thought the same way about the third person as the receiver. | 1.66 (0.09) | 3.86 | 1.87 |
| 1. I wanted to find out whether my ideas about the third person were accurate. | 1.52 (0.10) | 3.57 | 1.88 |
| Social enjoyment |  | 3.52 | 1.43 |
| 1. It was a pleasant pastime. | 0.79 (0.14) | 3.77 | 1.58 |
| 1. I wanted to have a good time with the recipient. | 1.35 (0.12) | 3.41 | 1.72 |
| 1. Talking like this was a pleasant activity. | 1.48 (0.10) | 3.75 | 1.69 |
| 1. I wanted to have fun spending time with the recipient. | 1.56 (0.09) | 3.41 | 1.72 |
| 1. I wanted to have a pleasant time with the recipient. | 1.57 (0.09) | 3.25 | 1.79 |
| Negative influence |  | 2.40 | 1.35 |
| 1. I wanted to damage the third person’s reputation. | 1.19 (0.09) | 2.06 | 1.49 |
| 1. I wanted to speak ill of the third person. | 1.22 (0.09) | 2.21 | 1.52 |
| 1. I wanted to negatively influence the recipient’s impression of the third person. | 1.39 (0.09) | 2.22 | 1.55 |
| 1. I wanted to discredit the third person. | 1.38 (0.08) | 2.13 | 1.48 |
| 1. I wanted to discuss the third person’s negative characteristics. | 1.22 (0.11) | 3.37 | 1.95 |
| Group protection |  | 2.96 | 1.62 |
| 1. I wanted to protect the recipient from the third person’s behavior. | 1.57 (0.10) | 2.66 | 1.85 |
| 1. I wanted to ensure that the recipient would know what kind of person the third person is. | 0.99 (0.14) | 3.66 | 1.91 |
| 1. I wanted to warn the receiver for the third person’ behavior. | 1.67 (0.10) | 2.89 | 1.93 |
| 1. I wanted to make sure that the recipient was not victimized by the third person’s behavior. | 1.75 (0.08) | 2.75 | 1.86 |
| 1. I wanted to prevent the recipient from becoming a victim of the third person’s behavior. | 1.77 (0.08) | 2.82 | 1.89 |

*Note.* Each item starts with the stem “For me, a reason to initiate this communication was …”. The latent factors were standardized, allowing free estimation of all factor loadings. All items were statistically significant at *p* < .001.

## Supplementary Table 4

Factor loadings and descriptive statistics for the MGQ in the simple definition condition.

| MGQ Factor | Standardized Loading (*SE*) | *M* | *SD* |
| --- | --- | --- | --- |
| Emotion venting |  | 4.00 | 1.62 |
| 1. I wanted to get something off my chest. | 1.43 (0.12) | 4.59 | 1.82 |
| 1. I wanted to vent. | 1.60 (0.11) | 3.70 | 1.99 |
| 1. I wanted to resolve my feelings by talking. | 1.65 (0.11) | 4.31 | 1.89 |
| 1. I wanted to share my feelings. | 1.63 (0.11) | 4.00 | 1.87 |
| 1. I hoped it would make me feel better. | 1.40 (0.13) | 3.29 | 1.97 |
| Information gathering and validation |  | 4.22 | 1.64 |
| 1. I wanted to find out if the recipient felt the same about the third person as I did. | 1.23 (0.14) | 4.52 | 1.76 |
| 1. I wanted to find out whether my impression of the third person was accurate. | 1.54 (0.12) | 4.14 | 1.99 |
| 1. I wanted to find out whether the recipient had the same ideas about the third person as I did. | 1.54 (0.11) | 4.41 | 1.81 |
| 1. I wanted to find out if I thought the same way about the third person as the receiver. | 1.57 (0.11) | 4.24 | 1.89 |
| 1. I wanted to find out whether my ideas about the third person were accurate. | 1.61 (0.11) | 4.17 | 1.92 |
| Social enjoyment |  | 4.07 | 1.42 |
| 1. It was a pleasant pastime. | 1.03 (0.13) | 3.42 | 1.71 |
| 1. I wanted to have a good time with the recipient. | 1.43 (0.10) | 3.39 | 1.74 |
| 1. Talking like this was a pleasant activity. | 1.16 (0.13) | 3.58 | 1.70 |
| 1. I wanted to have fun spending time with the recipient. | 1.53 (0.09) | 3.16 | 1.69 |
| 1. I wanted to have a pleasant time with the recipient. | 1.53 (0.10) | 3.10 | 1.74 |
| Negative influence |  | 2.52 | 1.29 |
| 1. I wanted to damage the third person’s reputation. | 1.15 (0.10) | 2.05 | 1.42 |
| 1. I wanted to speak ill of the third person. | 1.24 (010) | 2.13 | 1.49 |
| 1. I wanted to negatively influence the recipient’s impression of the third person. | 1.24 (0.09) | 2.34 | 1.55 |
| 1. I wanted to discredit the third person. | 1.41 (0.09) | 2.36 | 1.58 |
| 1. I wanted to discuss the third person’s negative characteristics. | 0.97 (0.08) | 3.75 | 1.97 |
| Group protection |  | 3.18 | 1.71 |
| 1. I wanted to protect the recipient from the third person’s behavior. | 1.63 (0.12) | 2.92 | 1.95 |
| 1. I wanted to ensure that the recipient would know what kind of person the third person is. | 1.10 (0.14) | 3.73 | 1.88 |
| 1. I wanted to warn the receiver for the third person’ behavior. | 1.79 (0.09) | 3.14 | 2.01 |
| 1. I wanted to make sure that the recipient was not victimized by the third person’s behavior. | 1.80 (0.09) | 3.05 | 1.97 |
| 1. I wanted to prevent the recipient from becoming a victim of the third person’s behavior. | 1.88 (0.08) | 3.06 | 1.98 |

*Note.* Each item starts with the stem “For me, a reason to initiate this communication was …”. The latent factors were standardized, allowing free estimation of all factor loadings. All items were statistically significant at *p* < .001.

## Supplementary Table 5

Correlations between the MGQ factors.

| MGQ Factor | 1 | 2 | 3 | 4 | 5 |
| --- | --- | --- | --- | --- | --- |
| 1. Emotion venting |  |  |  |  |  |
| 2. Information gathering and validation | .46 |  |  |  |  |
| 3. Social enjoyment | .22 | .18 |  |  |  |
| 4. Negative influence | .38 | .34 | .35 |  |  |
| 5. Group protection | .37 | .49 | .20 | .63 |  |

*Note.* All correlations were statistically significant at *p* < .001.

## Supplementary Table 6

Internal consistency (Cronbach’s α) overall and per condition and comparison of internal consistency between conditions.

|  |  |  |  |  | Condition comparison | |
| --- | --- | --- | --- | --- | --- | --- |
| Dimension | Overall | Broad | Narrow | Simple | χ^2^ (2) | *p* |
| Social enjoyment | .90 | .91 | .89 | .89 | 1.38 | .500 |
| Information gathering and validation | .91 | .90 | .92 | .90 | 1.72 | .423 |
| Negative influence | .88 | .89 | .90 | .86 | 2.97 | .227 |
| Group protection | .91 | .89 | .91 | .92 | 2.58 | .275 |
| Emotion venting | .90 | .90 | .91 | .90 | 0.40 | .827 |

*Note.* Comparisons made using the cocron package in R (Diedenhofen & Musch, 2016).

## Supplementary Table 7

Fit of the five-factor model per condition.

| Type of invariance | χ2 | *df* | Robust CFI | Robust TLI | Robust RMSEA | SRMR |
| --- | --- | --- | --- | --- | --- | --- |
| Overall | 744.40 | 265 | .926 | .916 | .073 | .079 |
| Broad | 478.56 | 265 | .913 | .902 | .082 | .091 |
| Narrow | 463.37 | 265 | .917 | .906 | .082 | .093 |
| Simple | 413.55 | 265 | .932 | .923 | .070 | .091 |

## Supplementary Table 8

Overview of changes from the original MGQ to the revised MGQ.

| Dimension | Original | Revised | Type of change |
| --- | --- | --- | --- |
| Information gathering and validation | to check whether the person I was talking with thought the same about the person we talked about. | I wanted to find out if the recipient felt the same about the third person as I did. | Reworded |
|  | to check whether my image of the person we talked about was correct. | I wanted to find out whether my impression of *the third person* was accurate. | Reworded |
|  | to get to know whether the person I was talking with had the same ideas as I had. | I wanted to find out whether *the recipient* had the same ideas about *the third person* as I did. | Reworded |
|  | to check whether the person I talked to had the same ideas about the person we talked about. | I wanted to find out if I thought the same way about the *third person* as *the recipient*. | Reworded |
|  | to check my opinion about the person we talked about. | - | Deleted |
|  | to find out whether the person I was talking with agreed with me. | - | Deleted |
|  | to learn whether the person I was talking with and I had the same views. | - | Deleted |
|  | to compare my ideas about the person we talked about with the person I talked with. | - | Deleted |
| Social enjoyment | to engage in an enjoyable activity. | It was a pleasant pastime. | Reworded |
|  | to engage in a fun activity with the person I was talking with. | Talking like this was a pleasant activity. | Reworded |
|  | to kill time with the person I was talking with. | I wanted to have fun spending time with *the recipient*. | Reworded |
|  | to have a good time. | I wanted to have a pleasant time with *the recipient*. | Reworded |
| Negative influence | to say negative things about the person we talked about. | I wanted to speak ill of *the third person*. | Reworded |
|  | to negatively influence the image that the person I was talking with has of the person we talked about. | I wanted to negatively influence *the recipient*’s impression of *the third person*. | Reworded |
|  | to put the person we talked about in a negative light. | I wanted to discredit *the third person*. | Reworded |
| Emotion Ventilation | - | I wanted to get something off my chest. | Added |
|  | - | I wanted to let off steam. | Added |
|  | - | I wanted to resolve my feelings by talking. | Added |
|  | - | I wanted to vent my emotions | Added |
|  | - | I hoped it would make me feel better. | Added |
|  | - | I wanted to reduce my negative feelings. | Deleted |
|  | - | I wanted to share my feelings. | Deleted |
| Group protection | to protect the person I was talking with against the person we were talking about. | I wanted to protect *the recipient* from *the third person*’s behavior | Reworded |
|  | to prevent that the person I was talking with would be exploited by the person we talked about. | I wanted to prevent *the recipient* from becoming a victim of *the third person*’s behavior. | reworded |
|  | **-** | I wanted to ensure that *the recipient* would know what kind of person *the third person* is. | Added |
|  | **-** | I wanted to make sure that *the recipient* was not victimized by *the third person*’s behavior. | Added |

# Gossip Definition Condition Instructions

## Broad Definition Instructions

In this study, we want to know how people communicate and share information about each other in their daily lives. Specifically, we want to know about situations when people communicate about others who are absent or have no knowledge of the communicated information.

We want you to recall and report about the most recent situation when you *communicated* (or *received*) information about another person, who was absent or unable to hear the conversation. Such information can relate to personal characteristics, attributes, events, behaviors, or needs of this other person.

In sum, the most important aspects of the situation we want you to report on are:

1. **(a) Person A communicates some personal information (such as personal characteristics, attributes, events, behaviors, or needs) about person C to person B.**
2. **(b) Person C must be absent (or not know of the information exchanged)**

In these communications, there are three persons involved:

(1) **THE SENDER** (person A): the person who communicated the information;

(2) **THE RECEIVER** (person B): the person who receives information;

(3) **THE TARGET** (person C): the person about whom information is communicated about.

You are either in the position of the sender (if you *communicated* the information) or the receiver (if you *received* the information).

The communication can be either spoken face-to-face, on the phone, or through emails, text or voice messages, other forms of social media, or any form of verbal communication. It can take place in any setting and with any number of people being present and/or involved in the interaction.

If the target, receiver, or sender included multiple people, we would like you to think of one specific person who represent each role, and then respond to questions with that person in mind.

The information that is communicated can range from general information about a person’s character to very specific information about how this person behaved in a situation. But importantly, the information must be about a specific person (i.e., the target). It is important to note that the information communicated about the target, can be positive, negative or simply factual and neutral (i.e. neither positive nor negative). Also, the information does not have to be something that remains a secret to the target.

## Narrow Definition Instructions

In this study, we want to know how people communicate and share information about each other in their daily lives. Specifically, we want to know about situations when people communicate about others who are absent or have no knowledge of the communicated information.

We want you to recall and report about a situation when you *communicated* (or *received*) personal characteristics, attributes, events, behaviors, or needs of another person, who was absent or unable to hear the conversation. We want you to recall and report about the most recent situation when you *communicated* (or *received*) **positive or negative** information about another person, who was absent or unable to hear the conversation. Such information can relate to personal characteristics, attributes, events, behaviors, or needs of this other person.

In sum, the most important aspects of the situation we want you to report on are:

1. **(a) Person A communicates positive or negative personal information (such as personal characteristics, attributes, events, behaviors, or needs) about person C to person B in an evaluative manner (positive or negative).**
2. **(b) The information must portray Person C in a positive or negative way**
3. **(c) Person C must be absent (or not know of the information exchanged)**

In these communications, there are three persons involved:

(1) **THE SENDER** (person A): the person who communicated the information;

(2) **THE RECEIVER** (person B): the person who receives information;

(3) **THE TARGET** (person C): the person about whom information is communicated about.

You are either in the position of the sender (if you *communicated* the information).

The communication can be either spoken face-to-face, on the phone, or through emails, text or voice messages, other forms of social media, or any form of verbal communication. It can take place in any setting and with any number of people being present and/or involved in the interaction.

If the target, receiver, or sender included multiple people, we would like you to think of one specific person who can represent each role, and then respond to questions with that person in mind.

The information that is communicated can range from general information about a person’s character to very specific information about how this person behaved in a situation. But importantly, the information must be about a specific person (i.e., the target). It is important to note that the information communicated about the target must be either positive and/or negative (i.e. not neutral). Also, the information does not have to be something that remains a secret to the target.

## Simple Definition Instructions

In this study, we want to know how people communicate and share information about each other in their daily lives. Specifically, we want to know about situations when people communicate about others who are absent or have no knowledge of the communicated information.

We want you to recall and report about a situation when you *communicated* (or *received*) gossip.

The most important aspects of the gossip that we want you to report on are:

1. **(a) Person A communicates some personal information (such as personal characteristics, attributes, events, behaviors, or needs) about person C to person B.**
2. **(b) Person C must be absent (or not know of the information exchanged)**

In these gossip events, there are three persons involved:

(1) **THE SENDER** (person A): the person who communicated the information;

(2) **THE RECEIVER** (person B): the person who receives information;

(3) **THE TARGET** (person C): the person about whom information is communicated about.

You are either in the position of the sender (if you *communicated* the information) or the receiver (if you *received* the information).

The gossip can be either spoken face-to-face, on the phone, or through emails, text or voice messages, other forms of social media, or any form of verbal communication.

If the target, receiver, or sender included multiple people, we would like you to think of one specific person who can represent each role, and then respond to questions with that person in mind.

The content of the gossip can be anything. But importantly, the information must be about specific person

# Motives to Gossip Questionnaire Instructions

We now present you with a number of possible reasons for initiating the communication you described above (the communication that took place in the task described). Please report to what extent each of the following was a reason for you to initiate communication.

For me, a reason to initiate this communication was …

# Complete Survey Dutch

Please see the separate pdf file in the supplementary materials (https://www.frontiersin.org/articles/10.3389/fpsyg.2019.01190/full#supplementary-material).
