# Supplementary material for: Getting a Grip on the Grapevine: Extension and Factor Structure of the Motives to Gossip Questionnaire
Source: Front Psychol. 2019 May 24;10:1190. doi: 10.3389/fpsyg.2019.01190 (PMC6543765; doi:10.3389/fpsyg.2019.01190)
Supplement: Supplementary file 2 [file Data_Sheet_1.PDF]

INFORMED CONSENT

Toestemmingsformulier

Beste Deelnemer,

U wordt gevraagd deel te nemen aan een onderzoek uitgevoerd door Dr. Daniel Balliet, Universitair Hoofddocent aan de Vrije Universiteit Amsterdam, en Terence das Dores Cruz onderzoeks-masterstudent aan de Vrije Universiteit Amsterdam. Dit formulier zal de gang van zaken tijdens het onderzoek toelichten en uitleg geven over de mogelijke risico's en voordelen.

Procedure

Als u instemt met deelname aan deze studie, zullen we u vragen om **een vragenlijst** in te vullen. Hierin zullen we u vragen om twee sociale situaties te beschrijven die u recent heeft meegemaakt met andere personen in uw dagelijks leven. Verder zullen we u een aantal vragen stellen over uw relatie met de andere personen en uw gedachtes over de situaties. Tenslotte vragen we u een schatting te maken hoe frequent deze situaties zijn in uw dagelijks leven.

Deelname aan dit onderzoek zal ongeveer **20 minuten** duren.

Vertrouwelijkheid

Uw deelname aan dit onderzoek is geheel anoniem en al uw antwoorden zullen vertrouwelijk blijven. Er worden geen namen of andere herleidbare gegevens vastgelegd tijdens deze studie. We vragen u niet naar uw naam, geboortedatum, adres of andere informatie die redelijkerwijs aan u te verbinden is. Eigenlijk is het moeilijk (al dan niet onmogelijk) om uw antwoorden aan u als individu te verbinden. Om documentatie van

wetenschappelijk werk te garanderen, wordt geanonimiseerde data uit dit onderzoek voor onbepaalde tijd opgeslagen na de publicatie van onze bevindingen. De geanonimiseerde data kan worden gedeeld met andere onderzoekers, en kan gebruikt worden voor onderzoeks- en onderwijsdoeleinden. Identificerende gegevens zullen nooit gebruikt worden in onze rapporten of publicatie, noch zal die informatie met anderen worden gedeeld.

Risico's

Er zijn geen verwachte risico's bij deelname aan deze studie. Hoewel het mogelijk is dat het beschrijven van situaties tijdelijk voor ongemak kan zorgen (als de situatie onaangenaam is), verwachten we geen ongemak buiten dat wat wordt ervaren in het dagelijks leven.

Voordelen

Deelname aan psychologisch onderzoek kan een leerzame ervaring zijn. Door deel te nemen, zou u kunnen leren over psychologische processen en draagt u bij aan wetenschappelijk onderzoek.

Vragen

Als u op elk moment vragen, zorgen, of klachten over het onderzoek heeft, kunt u contact opnemen met Terence das Dores Cruz (t.d.das.dorescruz@vu.nl), onderzoeks-master student aan de Vrije Universiteit Amsterdam, Nederland.

Toestemming

Als u 18 jaar of ouder bent, de bovenstaande informatie zorgvuldig heeft gelezen en begrepen heeft kunt u tekenen voor uw toestemming en starten met de studie.

○ Ik verklaar dat ik bovenstaande informatie gelezen en begrepen heb en geef mijn toestemming om deel te nemen aan dit onderzoek.

DEF - BROAD

In dit onderzoek willen we onderzoeken hoe mensen communiceren en informatie delen over elkaar in het dagelijks leven. Specifiek willen we situaties onderzoeken waar mensen communiceren over anderen, die afwezig zijn of geen kennis van de gedeelde informatie hebben.

We willen dat u zich de meeste recente situatie waarin u informatie *communiceerde* (of *ontving*) over een andere persoon herinnert en hierover verslag doet. Het gaat om een derde persoon die afwezig was of geen kennis had van de gedeelde informatie. Deze informatie kan gaan over persoonlijke kenmerken, eigenschappen, gebeurtenissen, gedrag, of behoeftes van deze derde persoon.

Kortom, de belangrijkste aspecten van de situatie waarover we willen dat u verslag geeft zijn:

1. **Persoon A communiceert aan Persoon B enige persoonlijke informatie over een derde persoon, Persoon C (bijvoorbeeld over persoonlijke kenmerken, eigenschappen, gebeurtenissen, gedrag, of behoeftes).**
2. **Persoon C moet afwezig zijn (of niets weten over de gedeelde informatie)**

In deze communicatie zijn dus ten minste drie personen betrokken:

1. **DE VERZENDER** (persoon A): de persoon die de informatie

communiceert;

2. **DE ONTVANGER** (persoon B): de persoon die de informatie ontvangt;
3. **DE DERDE PERSOON** (persoon C): de persoon over wie de informatie wordt gecommuniceerd

We zullen u vragen een situatie te herinneren waarin u de verzender was (als u de informatie *communiceerde*) en een situatie waarin u de ontvanger was (als u de informatie *ontving*).

De communicatie kan face-to-face gesproken, via de telefoon gesproken, of via e-mails, tekst of gesproken berichten, andere vormen van sociale media, of via elke andere vorm van verbale communicatie zijn. De communicatie kan plaatsvinden in elke omgeving, ongeacht het aantal personen dat aanwezig en/of betrokken is bij deze situatie.

Als de derde persoon, de ontvanger, of de verzender meerdere personen omvatten, vragen wij dat u één specifieke persoon in gedachten neemt en vervolgens de vragen beantwoordt met deze persoon (als ontvanger, verzender, of derde persoon) in gedachten.

De informatie die wordt gecommuniceerd kan variëren van algemene informatie over een persoons karakter tot hele specifieke informatie over hoe deze persoon zich gedroeg in een situatie. Maar, belangrijk is dat de informatie gaat over een specifiek persoon (d.w.z. een derde persoon). Het is van belang om in acht te nemen dat de informatie die gecommuniceerd wordt over de derde persoon positief, negatief, of simpelweg feitelijk en neutraal (d.w.z. positief noch negatief) kan zijn. Ook hoeft de informatie niet iets te zijn wat geheim blijft voor de derde persoon.

***** Uw deelname aan dit onderzoek is geheel anoniem en alle informatie die u geeft over de situaties blijft vertrouwelijk en kan niet achterhaald of naar u herleid worden. *****

DEF - NARROW

In dit onderzoek willen we onderzoeken hoe mensen communiceren en informatie delen over elkaar in het dagelijks leven. Specifiek willen we situaties onderzoeken waar mensen communiceren over anderen, die afwezig zijn of geen kennis van de gedeelde informatie hebben.

We willen dat u zich de meeste recente situatie waarin u **positieve of negatieve** informatie *communiceerde* (of *ontving*) over een andere person, die afwezig was of geen kennis had van de gedeelde informatie herinnert en hierover verslag geeft. Deze informatie kan gaan over persoonlijke kenmerken, eigenschappen, gebeurtenissen, gedrag, of behoeftes van die andere persoon.

Kortom, de belangrijkste aspecten van de situatie waarover we willen dat u verslag geeft zijn:

1. **Persoon A communiceert aan Persoon B enige positieve of negatieve persoonlijke informatie over een derde persoon, Persoon C (bijvoorbeeld over persoonlijke kenmerken, eigenschappen, gebeurtenissen, gedrag, of behoeftes).**
2. **De informatie moet persoon C positief of negatief afschilderen**
3. **Persoon C moet afwezig zijn (of niets weten over de gedeelde informatie)**

In deze communicatie zijn drie personen betrokken:

1. **DE VERZENDER** (persoon A): de persoon die de informatie communiceert;
2. **DE ONTVANGER** (persoon B): de persoon die de informatie ontvangt;
3. **DE DERDE PERSOON** (persoon C): de persoon over wie de informatie

wordt gecommuniceerd

We zullen u vragen een situatie te herinneren waarin u de verzender was (als u de informatie *communiceerde*) en een situatie waarin u de ontvanger was (als u de informatie *ontving*).

De communicatie kan face-to-face gesproken, via de telefoon gesproken, of via e-mails, tekst of gesproken berichten, andere vormen van sociale media, of via elke andere vorm van verbale communicatie zijn. De communicatie kan plaatsvinden in elke omgeving, ongeacht het aantal personen dat aanwezig en/of betrokken is bij deze situatie.

Als de derde persoon, de ontvanger, of de verzender meerdere personen omvatten, vragen wij dat u één specifieke persoon in gedachten neemt en vervolgens de vragen beantwoordt met deze persoon (als ontvanger, verzender, of derde persoon) in gedachten.

De informatie die wordt gecommuniceerd kan variëren van algemene informatie over een persoons karakter tot hele specifieke informatie over hoe deze persoon zich gedroeg in een situatie. Maar, belangrijk is dat de informatie gaat over een specifiek persoon (d.w.z. een derde persoon). Het is van belang om in acht te nemen dat de informatie die gecommuniceerd wordt over de derde persoon positief en/of negatief moet zijn (d.w.z. niet neutraal). Ook hoeft de informatie niet iets te zijn wat geheim blijft voor de derde persoon.

***** Uw deelname aan dit onderzoek is geheel anoniem en alle informatie die u geeft over de situaties blijft vertrouwelijk en kan niet achterhaald of naar u herleid worden. *****

DEF - GOSSIP

In dit onderzoek willen we onderzoeken hoe mensen roddelen in het dagelijks leven. Specifiek willen we situaties onderzoeken waar mensen communiceren over anderen, die afwezig zijn of geen kennis van de gedeelde informatie hebben.

We willen dat u zich de meeste recente situatie waarin u een roddel *communiceerde* (of *ontving*) over een andere persoon herinnert en hierover verslag doet.

De belangrijkste aspecten van het roddelen waarover we willen dat u verslag geeft zijn:

1. **Persoon A communiceert aan Persoon B enige persoonlijke informatie over een derde persoon, Persoon C (bijvoorbeeld over persoonlijke kenmerken, eigenschappen, gebeurtenissen, gedrag, of behoeftes).**
2. **Persoon C moet afwezig zijn (of niets weten over de gedeelde informatie).**

In deze roddel situaties zijn drie personen betrokken:

1. **DE VERZENDER** (persoon A): de persoon die de roddel communiceert;
2. **DE ONTVANGER** (persoon B): de persoon die de roddel ontvangt;
3. **DE DERDE PERSOON** (persoon C): de persoon over wie de roddel wordt gecommuniceerd

We zullen u vragen een situatie te herinneren waarin u de verzender was (als u de roddel *communiceerde*) en een situatie waarin u de ontvanger was (als u de roddel *ontving*).

De communicatie kan face-to-face gesproken, via de telefoon gesproken, of via e-mails, tekst of gesproken berichten, andere vormen van sociale media,

of via elke andere vorm van verbale communicatie zijn. Als de derde persoon, de ontvanger, of de verzender meerdere personen omvatten, vragen wij dat u één specifieke persoon in gedachten neemt en vervolgens de vragen beantwoordt met deze persoon (als ontvanger, verzender, of derde persoon) in gedachten.

De inhoud van de roddel kan alles zijn. Maar het is belangrijk dat de informatie over een specifieke persoon moet gaan.

***** Uw deelname aan dit onderzoek is geheel anoniem en alle informatie die u geeft over de situaties blijft vertrouwelijk en kan niet achterhaald of naar u herleid worden. *****

SENDING GOSSIP

Heeft u vandaag aan iemand informatie **gecommuniceerd** over een andere persoon, die geen kennis had over wat werd gecommuniceerd?

- ○ Ja (Herinner alstublieft de meest recente ervaring)
- ○ Nee

Heeft u vandaag aan iemand positieve of negatieve informatie **gecommuniceerd** over een andere persoon, die geen kennis had over wat werd gecommuniceerde?

- ○ Ja (Herinner alstublieft de meest recente ervaring)
- ○ Nee

Heeft u vandaag aan iemand een roddel **gecommuniceerd**?

- ○ Ja (Herinner alstublieft de meest recente ervaring)
- ○ Nee

	Uur	Minuten
Op welk tijdstip vond de communicatie plaats?		

We vragen u te denken aan de meeste recente situatie waarin u aan iemand informatie **communiceerde** over een andere persoon, die geen kennis had over wat er gecommuniceerd werd.

Wanneer vond deze communicatie plaats?

- ○ Vandaag
- ○ Gisteren
- ○ Twee dagen geleden
- ○ Drie dagen geleden
- ○ Vier dagen geleden
- ○ Vijf dagen geleden
- ○ Zes dagen geleden
- ○ Een week geleden
- ○ Meer dan een week geleden

We vragen u te denken aan de meeste recente situatie waarin u aan iemand positieve of negatieve informatie **communiceerde** over een andere persoon, die geen kennis had over wat er gecommuniceerd werd.

Wanneer vond deze communicatie plaats?

- Vandaag
- Gisteren
- Twee dagen geleden
- Drie dagen geleden
- Vier dagen geleden
- Vijf dagen geleden
- Zes dagen geleden
- Een week geleden
- Meer dan een week geleden

We vragen u te denken aan de meeste recente situatie waarin u aan iemand een roddel **communiceerde**.

Wanneer vond deze communicatie plaats?

- Vandaag
- Gisteren
- Twee dagen geleden
- Drie dagen geleden
- Vier dagen geleden
- Vijf dagen geleden
- Zes dagen geleden
- Een week geleden
- Meer dan week geleden

De volgende vragen hebben betrekking op **DE ONTVANGER**

De ontvanger is de persoon aan wie u de informatie communiceerde

Welke van de volgende opties beschrijft **de ontvanger**?

- Mijn partner
- Familielid
- Vriend
- Collega of Klasgenoot
- Leidinggevende of Instructeur/Leraar
- Kennis
- Vreemde
- Anders, namelijk... ________

Wat is het geslacht van **de ontvanger**?

- Man
- Vrouw
- Anders
- Weet ik niet

De volgende vragen hebben betrekking op **DE ONTVANGER**

De ontvanger is de persoon aan wie u de informatie communiceerde

Welke van de onderstaande stellingen beschrijft u relatie met **de ontvanger**.

U kunt waar kiezen voor meerdere stellingen

	Waar	Onwaar
Een persoon tot wie u zich zou wenden in tijden van ernstige		

emotionele of financiële zorgen	○	○
Een persoon, die het meest belangrijk voor u is en met wie voor altijd contact verliezen bedroevend zou zijn	○	○
Een persoon die niet in een van de bovenstaande beschrijving past, maar die nog steeds belangrijk voor u zou kunnen zijn en met wie u vaak contact heeft	○	○
Een persoon waarmee u vaak contact heeft maar wie niet belangrijk voor u is	○	○
Een persoon waarmee u zeer zelden contact heeft en wie niet belangrijk voor u is	○	○
Een persoon waar u nooit eerder contact mee heeft gehad	○	○

Hoeveel personen waren aanwezig en verkregen kennis over de gecommuniceerde informatie?

De volgende vragen hebben betrekking op **DE DERDE PERSOON**

De derde persoon is de persoon over wie de informatie werd gecommuniceerd

Welke van de volgende opties beschrijft **de derde persoon**?

- ○ Mijn partner
- ○ Familielid
- ○ Vriend
- ○ Collega of Klasgenoot
- ○ Leidinggevende of Instructeur/Leraar
- ○ Kennis
- ○ Vreemde

- Anders, namelijk... [ ]

Wat is het geslacht van **de derde persoon**?

- Man
- Vrouw
- Anders
- Ik weet het niet

De volgende vragen hebben betrekking op **DE DERDE PERSOON**

De derde persoon is de persoon over wie de informatie werd gecommuniceerd

Welke van de onderstaande stellingen beschrijft u relatie met **de derde persoon**.

U kunt waar kiezen voor meerdere stellingen

	Waar	Onwaar
Een persoon tot wie u zich zou wenden in tijden van ernstige emotionele of financiële zorgen	○	○
Een persoon, die het meest belangrijk voor u is en met wie voor altijd contact verliezen bedroevend zou zijn	○	○
Een persoon die niet in een van de bovenstaande beschrijving past, maar die nog steeds belangrijk voor u zou kunnen zijn en met wie u vaak contact heeft	○	○
Een persoon waarmee u vaak contact maar wie niet belangrijk voor u is	○	○
Een persoon waarmee u zeer zelden contact en wie niet belangrijk voor u is	○	○

Een persoon waar u nooit contact mee heeft	○	○
Een bekend persoon, die u niet heeft ontmoet (bijvoorbeeld een beroemdheid of een politicus)	○	○

Beschrijf alstublieft de informatie die u heeft gecommuniceerd in 1-3 zinnen

Waar heeft u de informatie die u communiceerde vandaan?

- Ik heb de informatie direct ervaren (het beïnvloedde mij)
- Ik observeerde hoe de informatie andere personen beïnvloedde
- Ik ontving de informatie van een andere persoon(personen), **die het direct ervaarde**
- Ik ontving de informatie van een andere persoon(personen), **die observeerde hoe het anderen beïnvloedde**
- Ik ontving de informatie van een andere persoon(personen), **die de informatie ontving van iemand anders**
- Ik ontving de informatie van een andere persoon(personen), **maar weet niet hoe zij kennis over de informatie verkregen**
- Ik heb de informatie verzonnen
- Ik weet het niet

Hoe heeft u de informatie gecommuniceerd?

- Face-to-face gesprek
- Online chat gesprek (bijvoorbeeld Whatsapp of Facebook Messenger)

- Sociale Media
- Email
- Opgenomen spraakbericht (bijvoorbeeld Whatsapp)
- Telefoon gesprek (online of offline)
- Media (televisie, krant, tijdschrift)
- Anders, namelijk...

De volgende vragen gaan over hoe de informatie **DE DERDE PERSOON** afschildert.

De derde persoon is de persoon over wie de informatie werd gecommuniceerd

Als u de mogelijkheid zou krijgen, zou u dan bereid zijn om de informatie direct aan **de derde persoon** te communiceren?

Helemaal niet bereid						Extreem bereid
○	○	○	○	○	○	○

Hoe positief was de informatie die u communiceerde over **de derde persoon**?

Helemaal niet positief						Extreem positief
○	○	○	○	○	○	○

Hoe negatief was de informatie die u communiceerde over **de derde persoon**?

Helemaal niet negatief						Extreem negatief
○	○	○	○	○	○	○

Ging de gedeelde informatie over het overtreden van een sociale norm (of regel) door **de derde persoon**?

- Ja
- Nee

De volgende vragen gaan over hoe de informatie **DE DERDE PERSOON** afschildert.

De derde persoon is de persoon over wie de informatie werd gecommuniceerd

Was de gedeelde informatie relevant met betrekking tot of **de derde persoon** KRACHTIG/DOMINANT versus ZWAK/ONDERDANIG is?

Helemaal niet relevant						Extreem relevant
○	○	○	○	○	○	○

Hoe **krachtig/dominant** is **de derde persoon** volgens de gedeelde informatie?

**Zwak/ Onderdanig**								**Krachtig/ Dominant**
○	○	○	○	○	○	○	○	○

Was de gedeelde informatie relevant met betrekking tot of **de derde persoon** WARM/VERDRAAGZAAM versus KOUD/ONVERDRAAGZAAM is?

Helemaal Extreem

niet relevant relevant

○ ○ ○ ○ ○ ○ ○

Hoe **warm/verdraagzaam** is **de derde persoon** volgens de gedeelde informatie?

Koud/ Onverdraagzaam **Warm/ Verdraagzaam**

○ ○ ○ ○ ○ ○ ○ ○ ○

Was de gedeelde informatie relevant met betrekking tot of **de derde persoon** BETROUWBAAR/EERLIJK versus ONBETROUWBAAR/ONEERLIJK is?

Helemaal niet relevant Extreem relevant

○ ○ ○ ○ ○ ○ ○

Hoe **betrouwbaar/eerlijk** is **de derde persoon** volgens de gedeelde informatie?

Onbetrouwbaar/ Oneerlijk **Betrouwbaar/ Eerlijk**

○ ○ ○ ○ ○ ○ ○ ○ ○

Was de gedeelde informatie relevant met betrekking tot of **de derde persoon** DESKUNDIG/COMPETENT versus ONWETEND/INCOMPETENT is?

Helemaal niet relevant Extreem relevant

○ ○ ○ ○ ○ ○ ○

Hoe **deskundig/competent** is **de derde persoon** volgens de gedeelde informatie?

Onwetend/ Incompetent ○ ○ ○ ○ ○ ○ ○ ○ ○ **Deskundig/ Competent**

Was de gedeelde informatie relevant met betrekking tot of **de derde persoon** MOREEL/ETHISCH versus IMMOREEL/ONETHISCH is?

Helemaal niet relevant ○ ○ ○ ○ ○ ○ ○ Extreem relevant

Hoe **moreel/ethisch** is **de derde persoon** volgens de gedeelde informatie?

Immoreel/ Onethisch ○ ○ ○ ○ ○ ○ ○ ○ ○ **Moreel/ Ethisch**

Was de gedeelde informatie relevant met betrekking tot of **de derde persoon** GEVOELIG/EMOTIONEEL versus ONGEVOELIG/EMOTIELOOS is?

Helemaal niet relevant ○ ○ ○ ○ ○ ○ ○ Extreem relevant

Hoe **gevoelig/emotioneel** is **de derde persoon** volgens de gedeelde informatie?

Ongevoelig/ **Gevoelig/**

**Emotieloos**								**Emotioneel**
○	○	○	○	○	○	○	○	○

Was de gedeelde informatie relevant met betrekking tot of **de derde persoon** SOCIAAL/EXTRAVERT versus GERESERVEERD/INTROVERT is?

Helemaal niet relevant						Extreem relevant
○	○	○	○	○	○	○

Hoe **sociaal/extravert** is **de derde persoon** volgens de gedeelde informatie?

**Gereserveerd/ Introvert**								**Sociaal/ Extravert**
○	○	○	○	○	○	○	○	○

Was de gedeelde informatie relevant met betrekking tot of **de derde persoon** VERANTWOORDELIJK/BEDACHTZAAM versus ONVERANTWOORDELIJK/ONBEDACHTZAAM is?

Helemaal niet relevant						Extreem relevant
○	○	○	○	○	○	○

Hoe **verantwoordelijk/bedachtzaam** is **de derde persoon** volgens de gedeelde informatie?

**Onverantwoordelijk/ Onbedachtzaam**								**Verantwoordelijk/ Bedachtzaam**
○	○	○	○	○	○	○	○	○

Was de gedeelde informatie relevant met betrekking tot of **de derde persoon** FANTASIERIJK/RUIMDENKEND versus FANTASIELOOS/BEKROMPEN is?

Helemaal niet relevant						Extreem relevant
○	○	○	○	○	○	○

Hoe **fantasierijk/ruimdenkend** is **de derde persoon** volgens de gedeelde informatie?

**Fantasieloos/ Bekrompen**								**Fantasierijk/ Ruimdenkend**
○	○	○	○	○	○	○	○	○

De volgende vragen hebben betrekking op uw relatie met **DE ONTVANGER**

De ontvanger is de persoon aan wie u de informatie communiceerde

Op dit moment voel ik me hecht of “close” **met de ontvanger**

Helemaal niet hecht						Extreem hecht
○	○	○	○	○	○	○

Wie heeft meer invloed in jullie relatie op dit moment?

De ontvanger			We zijn gelijk			Ik
○	○	○	○	○	○	○

Op dit moment is wat goed is voor **de ontvanger**, goed voor mij

Helemaal mee oneens	Grotendeels mee oneens	Enigszins mee oneens	Neutraal (niet mee eens noch mee oneens)	Enigszins mee eens	Grotendeels mee eens	Helemaal mee eens
○	○	○	○	○	○	○

Op dit moment ben ik toegewijd aan het behouden van mijn relatie met **de ontvanger**

Helemaal mee oneens	Grotendeels mee oneens	Enigszins mee oneens	Neutraal (niet mee eens noch mee oneens)	Enigszins mee eens	Grotendeels mee eens	Helemaal mee eens
○	○	○	○	○	○	○

Op dit moment vertrouw ik **de ontvanger**

Helemaal mee oneens	Grotendeels mee oneens	Enigszins mee oneens	Neutraal (niet mee eens noch mee oneens)	Enigszins mee eens	Grotendeels mee eens	Helemaal mee eens
○	○	○	○	○	○	○

De volgende vragen hebben betrekking op uw relatie met **DE ONTVANGER**

De ontvanger is de persoon aan wie u de informatie communiceerde

Hoe vaak heeft u contact met **de ontvanger**?

- Meerdere keren per dag
- Eens per dag
- Meerdere keren per week
- Eens per week
- Meerdere keren per maand
- Eens per maand
- Meerdere keren per jaar
- Eens per jaar
- Minder dan eens per jaar
- Nooit

De volgende vragen hebben betrekking op uw relatie met **DE ONTVANGER**

De ontvanger is de persoon aan wie u de informatie communiceerde

Hoeveel geld zou u op dit moment opgeven zodat **de ontvanger** 10 euro kan verdienen?

Ik zou .. euro opgeven

De volgende vragen hebben betrekking op uw relatie met **DE ONTVANGER**

De ontvanger is de persoon aan wie u de informatie communiceerde

Neutraal (niet mee

	Helemaal mee oneens	Grotendeels mee oneens	Enigszins mee oneens	eens noch mee oneens)	Enigszins mee eens	Grotendeel mee eens
Ik zou **de ontvanger** moeten confronteren	○	○	○	○	○	○
Ik zou **de ontvanger** moeten schaden	○	○	○	○	○	○
Ik zou **de ontvanger** moeten tegenwerken	○	○	○	○	○	○
Ik zou mijn uiterste best moeten doen om **de ontvanger** te helpen	○	○	○	○	○	○
Ik zou **de ontvanger** moeten vermijden	○	○	○	○	○	○
**De ontvanger** zou moeten worden uitgesloten van een groep waar ik bij hoor	○	○	○	○	○	○

De volgende vragen hebben betrekking op uw relatie met **DE DERDE PERSOON**

De derde persoon is de persoon over wie u de informatie communiceerde

Op dit moment voel ik me hecht of "close" met **de derde persoon**

Helemaal niet hecht						Extreem hecht
○	○	○	○	○	○	○

Wie heeft meer invloed in jullie relatie op dit moment?

De derde persoon			We zijn gelijk			Ik
○	○	○	○	○	○	○

Op dit moment is wat goed is voor **de derde persoon**, goed voor mij

Helemaal mee oneens	Grotendeels mee oneens	Enigszins mee oneens	Neutral	Enigszins mee eens	Grotendeels mee eens	Helemaal mee eens
○	○	○	○	○	○	○

Op dit moment ben ik toegewijd aan het behouden van mijn relatie met **de derde persoon**

Helemaal mee oneens	Grotendeels mee oneens	Enigszins mee oneens	Neutraal (niet mee eens noch mee oneens)	Enigszins mee eens	Grotendeels mee eens	Helemaal mee eens
○	○	○	○	○	○	○

Op dit moment vertrouw ik **de derde persoon**

Helemaal mee oneens	Grotendeels mee oneens	Enigszins mee oneens	Neutraal (niet mee eens noch mee oneens)	Enigszins mee eens	Grotendeels mee eens	Helemaal mee eens
○	○	○	○	○	○	○

De volgende vragen hebben betrekking op uw relatie met **DE DERDE PERSOON**

De derde persoon is de persoon over wie u de informatie communiceerde

Hoe vaak heeft u contact met **de derde persoon**?

- ○ Meerdere keren per dag
- ○ Eens per dag
- ○ Meerdere keren per week
- ○ Eens per week
- ○ Meerdere keren per maand
- ○ Eens per maand
- ○ Meerdere keren per jaar
- ○ Eens per jaar
- ○ Minder dan eens per jaar
- ○ Nooit

De volgende vragen hebben betrekking op uw relatie met **DE DERDE PERSOON**

De derde persoon is de persoon over wie u de informatie communiceerde

Hoeveel geld zou u op dit moment opgeven zodat **de derde persoon** 10 euro kan verdienen?

Ik zou .. euro opgeven

De volgende vragen hebben betrekking op uw relatie met **DE DERDE PERSOON**

De derde persoon is de persoon over wie u de informatie communiceerde

	Helemaal mee oneens	Grotendeels mee oneens	Enigszins mee oneens	Neutraal (niet mee eens noch mee oneens)	Enigszins mee eens	Grotendeel mee eens
Ik zou **de derde persoon** moeten confronteren	○	○	○	○	○	○
Ik zou **de derde persoon** moeten schaden	○	○	○	○	○	○
Ik zou **de derde persoon** moeten tegenwerken	○	○	○	○	○	○
Ik zou mijn uiterste best moeten						

doen om **de derde persoon** te helpen	○	○	○	○	○	○
Ik zou de **de derde persoon** moeten vermijden	○	○	○	○	○	○
**De derde persoon** zou moeten worden uitgesloten van een groep waar ik bij hoor	○	○	○	○	○	○

De volgende vragen hebben betrekking op de relatie tussen **DE ONTVANGER** en **DE DERDE PERSOON**

De ontvanger is de persoon aan wie u de informatie communiceerde

De derde persoon is de persoon over wie u de informatie communiceerde

Op dit moment zijn **de ontvanger** en **de derde persoon** hecht of "close"

Helemaal niet hecht						Extreem hecht
○	○	○	○	○	○	○

Wie heeft op dit moment meer invloed in de relatie tussen **de ontvanger** en **de derde persoon**?

De derde persoon			Ze zijn gelijk			De ontvanger
○	○	○	○	○	○	○

Op dit moment is wat goed is voor **de ontvanger**, goed voor **de derde persoon**

Helemaal mee oneens	Grotendeels mee oneens	Enigszins mee oneens	Neutral	Enigszins mee eens	Grotendeels mee eens	Helemaal mee eens
○	○	○	○	○	○	○

Op dit moment zijn **de ontvanger** en **de derde persoon** toegewijd aan het behouden van hun relatie

Helemaal mee oneens	Grotendeels mee oneens	Enigszins mee oneens	Neutraal (niet mee eens noch mee oneens)	Enigszins mee eens	Grotendeels mee eens	Helemaal mee eens
○	○	○	○	○	○	○

De volgende vragen hebben betrekking op de relatie tussen **DE ONTVANGER** en **DE DERDE PERSOON**

De ontvanger is de persoon aan wie u de informatie communiceerde

De derde persoon is de persoon over wie u de informatie communiceerde

Hoe vaak heeft **de ontvanger** contact met **de derde persoon**

- ○ Meerdere keren per dag
- ○ Eens per dag
- ○ Meerdere keren per week
- ○ Eens per week
- ○ Meerdere keren per maand
- ○ Eens per maand

- Meerdere keren per jaar
- Eens per jaar
- Minder dan eens per jaar
- Nooit

De volgende vragen hebben betrekking op de relatie tussen **DE ONTVANGER** en **DE DERDE PERSOON**

De ontvanger is de persoon aan wie u de informatie communiceerde

De derde persoon is de persoon over wie u de informatie communiceerde

	Helemaal mee oneens	Grotendeels mee oneens	Enigszins mee oneens	Neutraal (niet mee eens noch mee oneens)	Enigszins mee eens	Grotendeel mee eens
**De ontvanger** zou **de derde persoon** moeten confronteren	O	O	O	O	O	O
**De ontvanger** zou **de derde persoon** moeten schaden	O	O	O	O	O	O
**De ontvanger** zou **de**						

**derde persoon** moeten tegenwerken	○	○	○	○	○	○
**De ontvanger** zou zijn uiterste best moeten doen om **de derde persoon** te helpen	○	○	○	○	○	○
**De ontvanger** zou **de derde persoon** moeten vermijden	○	○	○	○	○	○
**De derde persoon** zou moeten worden uitgesloten van een groep waar **de ontvanger** bij hoort	○	○	○	○	○	○

MOTIVES

We leggen u nu een aantal mogelijke redenen voor om het gesprek dat u hierboven beschreef te beginnen. Wilt u hieronder steeds aangeven in hoeverre elk van deze redenen voor u een reden was om het gesprek te beginnen?

Ik begon deze communicatie omdat...

	Helemaal mee oneens	Grotendeels mee oneens	Enigszins mee oneens	Neutraal (niet mee eens noch mee oneens)	Enigszins mee eens	Grotende mee ee
het een prettige bezigheid was.	○	○	○	○	○	○
ik erachter wilde komen of **de ontvanger** hetzelfde dacht over **de derde persoon** als ik.	○	○	○	○	○	○
ik mijn gevoel wilde delen.	○	○	○	○	○	○
ik mijn ideeën over **de derde persoon** wilde vergelijken met die van **de ontvanger**.	○	○	○	○	○	○
ik de reputatie van **de derde persoon** wilde schaden.	○	○	○	○	○	○
ik mijn hart wilde luchten.	○	○	○	○	○	○

ik erachter wilde komen of mijn beeld van **de derde persoon** juist was.						
ik **de ontvanger** wilde beschermen tegen het gedrag van **de derde persoon**.						

	Helemaal mee oneens	Grotendeels mee oneens	Enigszins mee oneens	Neutraal (niet mee eens noch mee oneens)	Enigszins mee eens	Grotende mee ee
ik stoom wilde afblazen.						
ik een leuke tijd met **de ontvanger** wilde hebben.						
ik erachter wilde komen of **de ontvanger** dezelfde ideeën had over **de derde persoon** als ik.						
ik mijn gevoelens van me af wilde praten.						

ik kwaad wilde spreken over **de derde persoon**.	○	○	○	○	○	○
ik wilde achterhalen of ik hetzelfde over **de derde persoon** dacht als **de ontvanger**.	○	○	○	○	○	○
het praten een leuke activiteit was.	○	○	○	○	○	○
ik mijn emoties wilde ventileren.	○	○	○	○	○	○
	Helemaal mee oneens	Grotendeels mee oneens	Enigszins mee oneens	Neutraal (niet mee eens noch mee oneens)	Enigszins mee eens	Grotende mee ee
ik mijn ideeën over **de derde persoon** wilde toetsen.	○	○	○	○	○	○
ik wilde zorgen dat **de ontvanger** zou weten wat voor persoon **de derde persoon**.	○	○	○	○	○	○
ik de tijd op een leuke manier met						

**de ontvanger** wilde doorbrengen.	○	○	○	○	○	○
ik erachter wilde komen of **de ontvanger** het met me eens was.	○	○	○	○	○	○
ik hoopte dat ik me dan beter zou voelen.	○	○	○	○	○	○
ik **de ontvanger** wilde waarschuwen voor het gedrag van **de derde persoon**.	○	○	○	○	○	○
ik het beeld dat **de ontvanger** had van **de derde persoon** negatief wilde beïnvloeden.	○	○	○	○	○	○
ik **de derde persoon** in een negatief daglicht wilde stellen.	○	○	○	○	○	○
	Helemaal mee	Grotendeels mee	Enigszins mee	Neutraal (niet mee eens noch mee	Enigszins mee	Grotende

	oneens	oneens	oneens	oneens)	eens	mee ee
ik wilde zorgen dat **de ontvanger** niet het slachtoffer zou worden van het gedrag van **de derde persoon**.	◯	◯	◯	◯	◯	◯
ik de negatieve eigenschappen van **de derde persoon** wilde bespreken.	◯	◯	◯	◯	◯	◯
ik wilde achterhalen of **de ontvanger** en ik dezelfde opvattingen over **de derde persoon** hadden.	◯	◯	◯	◯	◯	◯
ik mijn negatieve gevoel wilde verminderen.	◯	◯	◯	◯	◯	◯
ik een gezellige tijd wilde hebben.	◯	◯	◯	◯	◯	◯
ik wilde voorkomen dat **de ontvanger** de dupe zou worden van het gedrag	◯	◯	◯	◯	◯	◯

van **de derde persoon**.						
ik erachter wilde komen of mijn ideeën over **de derde persoon** juist waren.	○	○	○	○	○	○
omdat ik informatie in ruil verwacht van **de ontvanger**.	○	○	○	○	○	○

	Helemaal mee oneens	Grotendeels mee oneens	Enigszins mee oneens	Neutraal (niet mee eens noch mee oneens)	Enigszins mee eens	Grotende mee ee
omdat ik informatie in ruil verwacht van anderen.	○	○	○	○	○	○
omdat ik een voordeel in ruil verwacht van **de ontvanger**.	○	○	○	○	○	○
omdat ik een voordeel in ruil verwacht van anderen.	○	○	○	○	○	○
omdat ik **de derde persoon** wil straffen.	○	○	○	○	○	○
omdat ik **de**						

**derde persoon** wil helpen.	○	○	○	○	○	○
omdat ik wil dat anderen **de derde persoon** straffen.	○	○	○	○	○	○
omdat ik wil dat anderen **de derde persoon** helpen.	○	○	○	○	○	○

FREQUENCY SENDING

Hoeveel andere situaties waar u informatie communiceerde over een andere persoon, die geen kennis had van de gecommuniceerde informatie, heeft u vandaag tussen 07.00 en 11.00 meegemaakt?

Tel alstublieft elke persoon waar u over communiceertde als een aparte gebeurtenis

Hoeveel andere situaties waar u informatie communiceerde over een andere persoon, die geen kennis had van de gecommuniceerde informatie, heeft u vandaag tussen 11.00 en 14.00 meegemaakt?

Tel alstublieft elke persoon waar u over communiceertde als een aparte gebeurtenis

Hoeveel andere situaties waar u informatie communiceerde over een andere

persoon, die geen kennis had van de gecommuniceerde informatie, heeft u vandaag tussen 14.00 en 18.00 meegemaakt?

Tel alstublieft elke persoon waar u over communiceertde als een aparte gebeurtenis

Hoeveel andere situaties waar u informatie communiceerde over een andere persoon, die geen kennis had van de gecommuniceerde informatie, heeft u **gisteren** tussen 17.00 en 23.00 meegemaakt?

Tel alstublieft elke persoon waar u over communiceertde als een aparte gebeurtenis

Hoeveel andere situaties waar u positieve/negatieve informatie communiceerde over een andere persoon, die geen kennis had van de gecommuniceerde informatie, heeft u vandaag tussen 07.00 en 11.00 meegemaakt?

Tel alstublieft elke persoon waar u over communiceertde als een aparte gebeurtenis

Hoeveel andere situaties waar u positieve/negatieve informatie communiceerde over een andere persoon, die geen kennis had van de gecommuniceerde informatie, heeft u vandaag tussen 11.00 en 14.00 meegemaakt?

Tel alstublieft elke persoon waar u over communiceertde als een aparte gebeurtenis

Hoeveel andere situaties waar u positieve/negatieve informatie communiceerde over een andere persoon, die geen kennis had van de gecommuniceerde informatie, heeft u vandaag tussen 14.00 en 18.00 meegemaakt?

Tel alstublieft elke persoon waar u over communiceertde als een aparte gebeurtenis

Hoeveel andere situaties waar u positieve/negatieve informatie communiceerde over een andere persoon, die geen kennis had van de gecommuniceerde informatie, heeft u **gisteren** tussen 17.00 en 23.00 meegemaakt?

Tel alstublieft elke persoon waar u over communiceertde als een aparte gebeurtenis

Hoeveel andere situaties waar u een roddel communiceerde heeft u vandaag tussen 07.00 en 11.00 meegemaakt?

Tel alstublieft elke persoon waar u over communiceertde als een aparte gebeurtenis

Hoeveel andere situaties waar u een roddel communiceerde heeft u vandaag tussen 11.00 en 14.00 meegemaakt?

Tel alstublieft elke persoon waar u over communiceertde als een aparte gebeurtenis

Hoeveel andere situaties waar u een roddel communiceerde heeft u vandaag tussen 14.00 en 18.00 meegemaakt?

Tel alstublieft elke persoon waar u over communiceertde als een aparte gebeurtenis

Hoeveel andere situaties waar u een roddel communiceerde heeft u **gisteren** tussen 17.00 en 23.00 meegemaakt?

Tel alstublieft elke persoon waar u over communiceertde als een aparte gebeurtenis

RECEIVING GOSSIP

Heeft u vandaag van iemand informatie **ontvangen** over een andere persoon, die geen kennis had over wat werd gecommuniceerd?

- Ja (Herinner alstublieft de meest recente ervaring)
- Nee

Heeft u vandaag van iemand positieve of negatieve informatie **ontvangen** over een andere persoon, die geen kennis had over wat werd gecommuniceerd?

- Ja (Herinner alstublieft de meest recente ervaring)
- Nee

Heeft u vandaag van iemand een roddel **ontvangen**?

- Ja (Herinner alstublieft de meest recente ervaring)
- Nee

	Uren	Minuten
Op welk tijdstip vond de communicatie plaats?		

We vragen u te denken aan de meeste recente situatie waarin u van iemand informatie **ontving** over een andere persoon, die geen kennis had over wat er gecommuniceerd werd.

Wanneer vond deze communicatie plaats?

- Vandaag
- Gisteren
- Twee dagen geleden
- Drie dagen geleden
- Vier dagen geleden
- Vijf dagen geleden
- Zes dagen geleden
- Een week geleden
- Meer dan een week geleden

We vragen u te denken aan de meeste recente situatie waarin u van iemand positieve of negatieve informatie **ontving** over een andere persoon, die geen

kennis had over wat er gecommuniceerd werd.

Wanneer vond deze communicatie plaats?

- Vandaag
- Gisteren
- Twee dagen geleden
- Drie dagen geleden
- Vier dagen geleden
- Vijf dagen geleden
- Zes dagen geleden
- Een week geleden
- Meer dan een week geleden

We vragen u te denken aan de meeste recente situatie waarin u van iemand een roddel **ontving**.

Wanneer vond deze communicatie plaats?

- Vandaag
- Gisteren
- Twee dagen geleden
- Drie dagen geleden
- Vier dagen geleden
- Vijf dagen geleden
- Zes dagen geleden
- Een week geleden
- Meer dan een week geleden

De volgende vragen hebben betrekking op **DE VERZENDER**

De verzender is de persoon die de informatie aan u communiceerde

Welke van de volgende opties beschrijft **de verzender**?

- ○ Mijn partner
- ○ Familielid
- ○ Vriend
- ○ Collega of Klasgenoot
- ○ Leidinggevende of Instructeur/Leraar
- ○ Kennis
- ○ Vreemde
- ○ Anders, namelijk... [ ]

Wat is het geslacht van **de verzender**?

- ○ Man
- ○ Vrouw
- ○ Anders
- ○ Ik weet het niet

De volgende vragen hebben betrekking op **DE VERZENDER**

De verzender is de persoon die de informatie aan u communiceerde

Welke van de onderstaande stellingen beschrijft u relatie met **de verzender**.

U kunt waar kiezen voor meerdere stellingen

	Waar	Onwaar

Een persoon tot wie u zich zou wenden in tijden van ernstige emotionele of financiële zorgen	○	○
Een persoon, die het meest belangrijk voor u is en met wie voor altijd contact verliezen bedroevend zou zijn	○	○
Een persoon die niet in een van de bovenstaande beschrijving past, maar die nog steeds belangrijk voor u zou kunnen zijn en met wie u vaak contact heeft	○	○
Een persoon waarmee u vaak contact heeft maar wie niet belangrijk voor u is	○	○
Een persoon waarmee u zeer zelden contact heeft en wie niet belangrijk voor u is	○	○
Een persoon waar u nooit eerder contact mee heeft gehad	○	○

Hoeveel personen waren aanwezig en verkregen kennis over de gecommuniceerde informatie?

[ ]

De volgende vragen hebben betrekking op **DE DERDE PERSOON**

De derde persoon is de persoon over wie de informatie werd gecommuniceerd

Welke van de volgende opties beschrijft **de derde persoon**?

- ○ Mijn partner
- ○ Familielid
- ○ Vriend
- ○ Collega of Klasgenoot
- ○ Leidinggevende of Instructeur/Leraar
- ○ Kennis

- Vreemde
- Anders, namelijk...

Wat is het geslacht van **de derde persoon**?

- Man
- Vrouw
- Anders
- Ik weet het niet

De volgende vragen hebben betrekking op **DE DERDE PERSOON**

De derde persoon is de persoon over wie de informatie werd gecommuniceerd

Welke van de onderstaande stellingen beschrijft u relatie met **de derde persoon**.

U kunt waar kiezen voor meerdere stellingen

	Waar	Onwaar
Een persoon tot wie u zich zou wenden in tijden van ernstige emotionele of financiële zorgen	○	○
Een persoon, die het meest belangrijk voor u is en met wie voor altijd contact verliezen bedroevend zou zijn	○	○
Een persoon die niet in een van de bovenstaande beschrijving past, maar die nog steeds belangrijk voor u zou kunnen zijn en met wie u vaak contact heeft	○	○
Een persoon waarmee u vaak contact heeft maar wie niet belangrijk voor u is	○	○

Een persoon waarmee u zeer zelden contact heeft en wie niet belangrijk voor u is	○	○
Een persoon waar u nooit contact mee heeft	○	○
Een bekend persoon, die u niet heeft ontmoet (bijvoorbeeld een beroemdheid of een politicus)	○	○

Beschrijf alstublieft de informatie die u heeft ontvangen in 1-3 zinnen

Hoe vaak heeft u deze informatie eerder ontvangen?

Hoeveel verschillende bronnen hebben deze informatie met u gecommuniceerd?

In hoeverre denkt u dat de informatie waar is?

Zeker onwaar				Ik weet het niet				Zeker waar
○	○	○	○	○	○	○	○	○

Waar heeft **de verzender** de informatie die hij/zij aan u communiceerde

vandaan?

- **De verzender** heeft de informatie direct ervaren (het beïnvloedde hem/haar)
- **De verzender** observeerde hoe de informatie andere personen beïnvloedde
- **De verzender** ontving de informatie van een andere persoon(personen), **die het direct ervaarde**
- **De verzender** ontving de informatie van een andere persoon(personen), **die observeerde hoe het anderen beïnvloedde**
- **De verzender** ontving de informatie van een andere persoon(personen), **die de informatie ontving van iemand anders**
- **De verzender** ontving de informatie van een andere persoon(personen**), maar weet niet hoe zij kennis over de informatie verkregen**
- Ik weet het niet

Hoe werd de informatie aan u gecommuniceerd?

- Face-to-face gesprek
- Online chat gesprek (bijvoorbeeld Whatsapp of Facebook Messenger)
- Sociale Media
- Email
- Opgenomen spraakbericht (bijvoorbeeld Whatsapp)
- Telefoon gesprek (online of offline)
- Media (televisie, krant, tijdschrift)
- Anders, namelijk... ________

De volgende vragen gaan over hoe de informatie **DE DERDE PERSOON** afschildert.

De derde persoon is de persoon over wie de informatie werd gecommuniceerd

Als u de mogelijkheid zou krijgen, zou u dan bereid zijn om de informatie

direct aan **de derde persoon** te communiceren?

Helemaal niet bereid						Extreem bereid
○	○	○	○	○	○	○

Hoe positief was de informatie die gecommuniceerd werd over **de derde persoon**?

Helemaal niet positief						Extreem positief
○	○	○	○	○	○	○

Hoe negatief was de informatie die gecommuniceerd werd over **de derde persoon**?

Helemaal niet negatief						Extreem negatief
○	○	○	○	○	○	○

Ging de informatie over het overtreden van een sociale norm (of regel) door **de derde persoon**?

○ Ja

○ Nee

De volgende vragen gaan over hoe de informatie **DE DERDE PERSOON** afschildert.

De derde persoon is de persoon over wie de informatie werd gecommuniceerd

Was de gedeelde informatie relevant met betrekking tot of **de derde persoon** KRACHTIG/DOMINANT versus ZWAK/ONDERDANIG is?

Helemaal niet relevant						Extreem relevant
○	○	○	○	○	○	○

Hoe **krachtig/dominant** is **de derde persoon** volgens de gedeelde informatie?

**Zwak/ Onderdanig**								**Krachtig/ Dominant**
○	○	○	○	○	○	○	○	○

Was de gedeelde informatie relevant met betrekking tot of **de derde persoon** WARM/VERDRAAGZAAM versus KOUD/ONVERDRAAGZAAM is?

Helemaal niet relevant						Extreem relevant
○	○	○	○	○	○	○

Hoe **warm/verdraagzaam** is **de derde persoon** volgens de gedeelde informatie?

**Koud/ Onverdraagzaam**								**Warm/ Verdraagzaam**
○	○	○	○	○	○	○	○	○

Was de gedeelde informatie relevant met betrekking tot of **de derde persoon** BETROUWBAAR/EERLIJK versus ONBETROUWBAAR/ONEERLIJK is?

Helemaal niet relevant						Extreem relevant
○	○	○	○	○	○	○

Hoe **betrouwbaar/eerlijk** is **de derde persoon** volgens de gedeelde informatie?

**Onbetrouwbaar/ Oneerlijk**								**Betrouwbaar/ Eerlijk**
○	○	○	○	○	○	○	○	○

Was de gedeelde informatie relevant met betrekking tot of **de derde persoon** DESKUNDIG/COMPETENT versus ONWETEND/INCOMPETENT is?

Helemaal niet relevant						Extreem relevant
○	○	○	○	○	○	○

Hoe **deskundig/competent** is **de derde persoon** volgens de gedeelde informatie?

**Onwetend/ Incompetent**								**Deskundig/ Competent**
○	○	○	○	○	○	○	○	○

Was de gedeelde informatie relevant met betrekking tot of **de derde persoon** MOREEL/ETHISCH versus IMMOREEL/ONETISCH is?

Helemaal niet relevant						Extreem relevant
○	○	○	○	○	○	○

Hoe **moreel/ethisch** is **de derde persoon** volgens de gedeelde informatie?

Immoreel/ Onethisch ○ ○ ○ ○ ○ ○ ○ ○ ○ **Moreel/ Ethisch**

Was de gedeelde informatie relevant met betrekking tot of **de derde persoon** GEVOELIG/EMOTIONEEL versus ONGEVOELIG/EMOTIELOOS is?

Helemaal niet relevant ○ ○ ○ ○ ○ ○ ○ Extreem relevant

Hoe **gevoelig/emotioneel** is **de derde persoon** volgens de gedeelde informatie?

Ongevoelig/ Emotieloos ○ ○ ○ ○ ○ ○ ○ ○ ○ **Gevoelig/ Emotioneel**

Was de gedeelde informatie relevant met betrekking tot of **de derde persoon** SOCIAAL/EXTRAVERT versus GERESERVEERD/INTROVERT is?

Helemaal niet relevant ○ ○ ○ ○ ○ ○ ○ Extreem relevant

Hoe **sociaal/extravert** is **de derde persoon** volgens de gedeelde informatie?

Gereserveerd/ **Sociaal/**

Introvert **Extravert**

Was de gedeelde informatie relevant met betrekking tot of **de derde persoon** VERANTWOORDELIJK/BEDACHTZAAM versus ONVERANTWOORDELIJK/ONBEDACHTZAAM is?

Helemaal niet relevant Extreem relevant

Hoe **verantwoordelijk/bedachtzaam** is **de derde persoon** volgens de gedeelde informatie?

Onverantwoordelijk/ Onbedachtzaam **Verantwoordelijk/ Bedachtzaam**

Was de gedeelde informatie relevant met betrekking tot of **de derde persoon** FANTASIERIJK/RUIMDEKEND versus FANTASIELOOS/BEKROMPEN is?

Helemaal niet relevant Extreem relevant

Hoe **fantasierijk/ruimdenkend** is **de derde persoon** volgens de gedeelde informatie?

Fantasieloos/ Bekrompen **Fantasierijk/ Ruimdenkend**

De volgende vragen hebben betrekking op uw relatie met **DE VERZENDER**

De verzender is de persoon die de informatie aan u communiceerde

Op dit moment voel ik me hecht of "close" met **de verzender**

Helemaal niet hecht						Extreem hecht
○	○	○	○	○	○	○

Wie heeft meer invloed in jullie relatie op dit moment?

De verzender			We zijn gelijk			Ik
○	○	○	○	○	○	○

Op dit moment is wat goed is voor **de verzender**, goed voor mij

Helemaal niet mee eens	Grotendeels mee oneens	Enigszins mee oneens	Neutraal (niet mee oneens noch mee eens)	Enigszins mee eens	Grotendeels mee eens	Helemaal mee eens
○	○	○	○	○	○	○

Op dit moment ben ik toegewijd aan het behouden van mijn relatie met **de verzender**

Helemaal			Neutraal (niet mee oneens

niet mee eens	Grotendeels mee oneens	Enigszins mee oneens	noch mee eens)	Enigszins mee eens	Grotendeels mee eens	Helemaal mee eens
○	○	○	○	○	○	○

Op dit moment vertrouw ik **de verzender**

Helemaal niet mee eens	Grotendeels mee oneens	Enigszins mee oneens	Neutraal (niet mee oneens noch mee eens)	Enigszins mee eens	Grotendeels mee eens	Helemaal mee eens
○	○	○	○	○	○	○

Hoe sympathiek is **de verzender** op dit moment?

Helemaal niet sympathiek						Extreem sympathiek
○	○	○	○	○	○	○

Hoe zou u de status van **de verzender** op dit moment beoordelen?

Lage status						Hoge status
○	○	○	○	○	○	○

De volgende vragen hebben betrekking op uw relatie met **DE VERZENDER**

De verzender is de persoon die de informatie aan u communiceerde

Hoe vaak heeft u contact met **de verzender**?

○ Meerdere keren per dag

- Eens per dag
- Meerdere keren per week
- Eens per week
- Meerdere keren per maand
- Eens per maand
- Meerdere keren per jaar
- Eens per jaar
- Minder dan eens per jaar
- Nooit

De volgende vragen hebben betrekking op uw relatie met **DE VERZENDER**

De verzender is de persoon die de informatie aan u communiceerde

Hoeveel geld zou u op dit moment opgeven zodat **de verzender** 10 euro kan verdienen?

Ik zou .. euro opgeven

De volgende vragen hebben betrekking op uw relatie met **DE VERZENDER**

De verzender is de persoon die de informatie aan u communiceerde

	Helemaal	Grotendeels	Enigszins	Neutraal (niet mee oneens noch	Enigszins

	niet mee eens	mee oneens	mee oneens	mee eens)	mee eens	Grotendeel mee eens
Ik zou **de verzender** moeten confronteren	○	○	○	○	○	○
Ik zou **de verzender** moeten schaden	○	○	○	○	○	○
Ik zou **de verzender** moeten tegenwerken	○	○	○	○	○	○
Ik zou mijn uiterste best moeten doen om **de verzender** te helpen	○	○	○	○	○	○
Ik zou **de verzender** moeten vermijden	○	○	○	○	○	○
**De verzender** zou moeten worden uitgesloten van een groep waar ik bij hoor	○	○	○	○	○	○

De volgende vragen hebben betrekking op uw relatie met **DE DERDE PERSOON**

De derde persoon is de persoon over wie de informatie werd gecommuniceerd

Op dit moment voel ik me hecht of "close" met **de derde persoon**

Helemaal niet hecht						Extreem hecht
○	○	○	○	○	○	○

Wie heeft meer invloed in jullie relatie op dit moment?

De derde persoon			We zijn gelijk			Ik
○	○	○	○	○	○	○

Op dit moment is wat goed is voor **de derde persoon**, goed voor mij

Helemaal niet mee eens	Grotendeels mee oneens	Enigszins mee oneens	Neutraal (niet mee oneens noch mee eens)	Enigszins mee eens	Grotendeels mee eens	Helemaal mee eens
○	○	○	○	○	○	○

Op dit moment ben ik toegewijd aan het behouden van mijn relatie met **de derde persoon**

Helemaal niet mee eens	Grotendeels mee oneens	Enigszins mee oneens	Neutraal (niet mee oneens noch mee eens)	Enigszins mee eens	Grotendeels mee eens	Helemaal mee eens
○	○	○	○	○	○	○

Op dit moment vertrouw ik **de derde persoon**

Helemaal niet mee eens	Grotendeels mee oneens	Enigszins mee oneens	Neutraal (niet mee oneens noch mee eens)	Enigszins mee eens	Grotendeels mee eens	Helemaal mee eens
○	○	○	○	○	○	○

Hoe sympathiek is **de derde persoon** op dit moment?

Helemaal niet sympathiek						Extreem sympathiek
○	○	○	○	○	○	○

Hoe zou u de status van **de derde persoon** op dit moment beoordelen?

Lage status						Hoge status
○	○	○	○	○	○	○

De volgende vragen hebben betrekking op uw relatie met **DE DERDE PERSOON**

De derde persoon is de persoon over wie de informatie werd gecommuniceerd

Hoe vaak heeft u contact met **de derde persoon**?

- ○ Meerdere keren per dag
- ○ Eens per dag
- ○ Meerdere keren per week
- ○ Eens per week
- ○ Meerdere keren per maand
- ○ Eens per maand
- ○ Meerdere keren per jaar

- Eens per jaar
- Minder dan eens per jaar
- Nooit

De volgende vragen hebben betrekking op uw relatie met **DE DERDE PERSOON**

De derde persoon is de persoon over wie de informatie werd gecommuniceerd

Hoeveel geld zou u op dit moment opgeven zodat **de derde persoon** 10 euro kan verdienen?

Ik zou .. euro opgeven

De volgende vragen hebben betrekking op uw relatie met **DE DERDE PERSOON**

De derde persoon is de persoon over wie de informatie werd gecommuniceerd

	Helemaal niet mee eens	Grotendeels mee oneens	Enigszins mee oneens	Neutraal (niet mee oneens noch mee eens)	Enigszins mee eens	Grotendeel mee eens
Ik zou **de derde persoon** moeten confronteren	○	○	○	○	○	○

Ik zou **de derde persoon** moeten schaden	○	○	○	○	○	○
Ik zou **de derde persoon** moeten tegenwerken	○	○	○	○	○	○
Ik zou mijn uiterste best moeten doen om **de derde persoon** te helpen	○	○	○	○	○	○
Ik zou **de derde persoon** moeten vermijden	○	○	○	○	○	○
**De derde persoon** zou moeten worden uitgesloten van een groep waar ik bij hoor	○	○	○	○	○	○

De volgende vragen hebben betrekking op de relatie tussen **DE VERZENDER** en **DE DERDE PERSOON**

De verzender is de persoon die de informatie aan u communiceerde

De derde persoon is de persoon over wie de informatie werd gecommuniceerd

Op dit moment zijn **de verzender** en **de derde persoon** hecht of “close”

Helemaal niet close						Extreem hecht
○	○	○	○	○	○	○

Wie heeft op dit moment meer invloed in de relatie tussen **de verzender** en **de derde persoon**?

De derde persoon			Ze zijn gelijk			De verzender
○	○	○	○	○	○	○

Op dit moment is wat goed is voor **de verzender**, goed voor **de derde persoon**

Helemaal niet mee eens	Grotendeels mee oneens	Enigszins mee oneens	Neutraal (niet mee oneens noch mee eens)	Enigszins mee eens	Grotendeels mee eens	Helemaal mee eens
○	○	○	○	○	○	○

Op dit moment zijn **de verzender** en **de derde persoon** toegewijd aan het behouden van hun relatie

Helemaal niet mee eens	Grotendeels mee oneens	Enigszins mee oneens	Neutraal (niet mee oneens noch mee eens)	Enigszins mee eens	Grotendeels mee eens	Helemaal mee eens
○	○	○	○	○	○	○

De volgende vragen hebben betrekking op de relatie tussen **DE**

VERZENDER en **DE DERDE PERSOON**

De verzender is de persoon die de informatie aan u communiceerde

De derde persoon is de persoon over wie de informatie werd gecommuniceerd

Hoe vaak heeft **de verzender** contact met **de derde persoon**?

- Meerdere keren per dag
- Eens per dag
- Meerdere keren per dag
- Eens per week
- Meerdere keren per maand
- Eens per maand
- Meerdere keren per jaar
- Eens per jaar
- Minder dan eens per jaar
- Nooit

De volgende vragen hebben betrekking op de relatie tussen **DE VERZENDER** en **DE DERDE PERSOON**

De verzender is de persoon die de informatie aan u communiceerde

De derde persoon is de persoon over wie de informatie werd gecommuniceerd

	Helemaal niet mee eens	Grotendeels mee oneens	Enigszins mee oneens	Neutraal (niet mee oneens noch mee eens)	Enigszins mee eens	Grotendeel mee eens

**De verzender** zou **de derde persoon** moeten confronteren	○	○	○	○	○	○
**De verzender** zou **de derde persoon** moeten schaden	○	○	○	○	○	○
**De verzender** zou **de derde persoon** moeten tegenwerken	○	○	○	○	○	○
**De verzender** zou zijn uiterste best moeten doen om **de derde persoon** te helpen	○	○	○	○	○	○
**De verzender** zou **de derde persoon** moeten vermijden	○	○	○	○	○	○
**De derde persoon** zou moeten worden uitgesloten van een	○	○	○	○	○	○

groep waar
de
verzender
bij hoort

FREQUENCY RECEIVING

Hoeveel andere situaties waar u informatie ontving over een andere persoon, die geen kennis had van de gecommuniceerde informatie, heeft u vandaag tussen 07.00 en 11.00 meegemaakt?

Tel alstublieft elke persoon waarover aan u over gecommuniceerd werd als een aparte gebeurtenis

Hoeveel andere situaties waar u informatie ontving over een andere persoon, die geen kennis had van de gecommuniceerde informatie, heeft u vandaag tussen 11.00 en 14.00 meegemaakt?

Tel alstublieft elke persoon waarover aan u over gecommuniceerd werd als een aparte gebeurtenis

Hoeveel andere situaties waar u informatie ontving over een andere persoon, die geen kennis had van de gecommuniceerde informatie, heeft u vandaag tussen 14.00 en 18.00 meegemaakt?

Tel alstublieft elke persoon waarover aan u over gecommuniceerd werd als een aparte gebeurtenis

Hoeveel andere situaties waar u informatie ontving over een andere persoon,

die geen kennis had van de gecommuniceerde informatie, heeft u **gisteren** tussen 17.00 en 23.00 meegemaakt?

Tel alstublieft elke persoon waarover aan u over gecommuniceerd werd als een aparte gebeurtenis

Hoeveel andere situaties waar u positieve of negatieve informatie ontving over een andere persoon, die geen kennis had van de gecommuniceerde informatie, heeft u vandaag tussen 07.00 en 11.00 meegemaakt?

Tel alstublieft elke persoon waarover aan u over gecommuniceerd werd als een aparte gebeurtenis

Hoeveel andere situaties waar u positieve of negatieve informatie ontving over een andere persoon, die geen kennis had van de gecommuniceerde informatie, heeft u vandaag tussen 11.00 en 14.00 meegemaakt?

Tel alstublieft elke persoon waarover aan u over gecommuniceerd werd als een aparte gebeurtenis

Hoeveel andere situaties waar u positieve of negatieve informatie ontving over een andere persoon, die geen kennis had van de gecommuniceerde informatie, heeft u vandaag tussen 14.00 en 18.00 meegemaakt?

Tel alstublieft elke persoon waarover aan u over gecommuniceerd werd als een aparte gebeurtenis

Hoeveel andere situaties waar u positieve of negatieve informatie ontving over een andere persoon, die geen kennis had van de gecommuniceerde

informatie, heeft u **gisteren** tussen 17.00 en 23.00 meegemaakt?

Tel alstublieft elke persoon waarover aan u over gecommuniceerd werd als een aparte gebeurtenis

Hoeveel andere situaties waar u een roddel ontving heeft u vandaag tussen 07.00 en 11.00 meegemaakt?

Tel alstublieft elke persoon waarover aan u over gecommuniceerd werd als een aparte gebeurtenis

Hoeveel andere situaties waar u een roddel ontving heeft u vandaag tussen 11.00 en 14.00 meegemaakt?

Tel alstublieft elke persoon waarover aan u over gecommuniceerd werd als een aparte gebeurtenis

Hoeveel andere situaties waar u een roddel ontving heeft u vandaag tussen 14.00 en 18.00 meegemaakt?

Tel alstublieft elke persoon waarover aan u over gecommuniceerd werd als een aparte gebeurtenis

Hoeveel andere situaties waar u een roddel ontving heeft u **gisteren** tussen 17.00 en 23.00 meegemaakt?

Tel alstublieft elke persoon waarover aan u over gecommuniceerd werd als een aparte gebeurtenis

FINAL QUESTION

Vonden de twee beschrijvingen die u heeft verstrekt over het communiceren en ontvangen van informatie over een andere persoon (die geen kennis had van de gecommuniceerde informatie) plaats in dezelfde situatie, met communicatie tussen dezelfde mensen?

- Ja, deze vonden plaats in dezelfde situatie
- Nee, het waren verschillende situaties

Vonden de twee beschrijvingen die u heeft verstrekt over het communiceren en ontvangen van positieve of negatieve informatie over een andere persoon (die geen kennis had van de gecommuniceerde informatie) plaats in dezelfde situatie, met communicatie tussen dezelfde mensen?

- Ja, deze vonden plaats in dezelfde situatie
- Nee, het waren verschillende situaties

Vonden de twee beschrijvingen die u heeft verstrekt over het communiceren en ontvangen van een roddel plaats in dezelfde situatie, met communicatie tussen dezelfde mensen?

- Ja, deze vonden plaats in dezelfde situatie
- Nee, het waren verschillende situaties

Powered by Qualtrics
